# Do meaningful dimensions of childhood adversity exist? Data‐driven evidence from two prospective cohort studies

**DOI:** 10.1111/jcpp.14098

**Published:** 2024-12-17

**Authors:** Athena R.W. Chow, Jessie R. Baldwin, Lucy Bowes

**Affiliations:** ^1^ Department of Experimental Psychology, Medical Sciences Division University of Oxford Oxford UK; ^2^ Division of Psychology and Language Sciences, Department of Clinical, Educational and Health Psychology University College London London UK; ^3^ Social, Genetic and Developmental Psychiatry Centre, Institute of Psychiatry, Psychology and Neuroscience King's College London London UK

**Keywords:** Adverse childhood experiences, victimisation, data‐driven methods, psychopathology, adolescence

## Abstract

**Background:**

There is not yet a consensus on the best way to conceptualise adverse childhood experiences (ACEs). We used data‐driven methods across two populations to examine (a) if there were meaningful dimensions underlying ACEs and (b) whether dimensions were differentially associated with increased risk of adolescent psychopathology.

**Methods:**

Participants were 18,539 British children from the UK Millennium Cohort Study (MCS) and 11,876 American children from the US Adolescent Brain Cognitive Development Study (ABCD). A wide range of ACEs (e.g., abuse, neglect, parental psychopathology, peer victimisation) were measured prospectively from infancy to mid‐adolescence using interviews and questionnaires. Internalising and externalising symptoms were assessed with child and/or parent reports during adolescence.

**Results:**

Our preregistered exploratory factor analysis revealed four latent dimensions in the MCS (parental threat, deprivation, victimisation, and parental discipline) and ABCD (parental threat, deprivation, victimisation, and traumatic events). All dimensions except deprivation were associated with increased risk for internalising and externalising symptoms. Over and above the other dimensions, victimisation was more strongly associated with internalising (MCS β = .34, 95% CI 0.33–0.36; ABCD β = .11, 95% CI 0.10–0.13) and externalising (MCS β = .31, 95% CI 0.30–0.33; ABCD β = .13, 95% CI 0.11–0.15) symptoms.

**Conclusions:**

Across two distinct populations, we found that ACEs can be captured by common underlying dimensions of parental threat, deprivation, and victimisation, as well as additional sample‐specific dimensions. Our findings expand dimensional theories of childhood adversity by suggesting that in addition to threat and deprivation, victimisation is a distinct dimension of adversity that has the strongest associations with adolescent psychopathology.

## Introduction

Adverse childhood experiences (ACEs) are globally associated with higher risks for psychopathology, chronic health diseases, and substance use throughout the life course (Bellis et al., 2019; Felitti et al., 1998; Hughes et al., 2017). Annual health costs associated with ACEs in North America and Europe are estimated to be $748 billion and $581 billion, respectively (Bellis et al., 2019). Despite the wealth of research that has established robust associations between ACEs and poor health, there is not yet a consensus across the field of developmental psychopathology on how ACEs should be conceptualised. Specifically, studies use inconsistent approaches to conceptualise ACEs, focus on different combinations of ACEs, and tend to follow different definitions of adversity (Lacey & Minnis, 2020).

Most research has followed the pioneering methodology of the CDC‐Kaiser ACE Study, which summed 10 ACEs as a cumulative risk score to demonstrate a dose–response relationship between multiple adversities and detrimental health outcomes (Baldwin et al., 2021; Felitti et al., 1998; Hughes et al., 2017). These 10 ACEs included abuse (emotional, physical, or sexual), neglect (emotional, or physical), and household dysfunction (parental marital discord, domestic violence, substance abuse, mental illness, or criminal behaviour). Subsequent research has incorporated more diverse ACEs; for instance, the World Health Organisation includes parental bereavement and exposure to peer, community, and collective violence (WHO, 2018). Research has also shown that expanding the original ACE scale to include peer victimisation, community violence, and low socioeconomic status (SES) significantly improved the prediction of mental and physical health problems (Finkelhor, Shattuck, Turner, & Hamby, 2015; Mersky, Janczewski, & Topitzes, 2017). Therefore, broadening the definition of adversity beyond the original ten ACEs could advance our understanding of the relationship between adversity and psychopathology.

While the cumulative risk approach has proven useful in establishing the dose–response relationship, it has two key limitations. First, by summing ACEs together, it implicitly assumes that different ACEs have equal effects on psychopathology; this is unlikely considering how heterogeneous ACEs are in severity, frequency, and duration. For example, sexual abuse and parental divorce are likely to impact physical and mental health outcomes in differential ways (Westermair et al., 2018). Second, as it lacks specificity in identifying the mechanisms through which ACEs impact development, the cumulative risk approach falls short in explaining how ACEs increase the risk for psychopathology. Recent studies have attempted to overcome these limitations by using dimensional models to conceptualise adversity. The Dimensional Model of Adversity and Psychopathology (DMAP) proposes that ACEs reflecting dimensions of threat (e.g., abuse) and deprivation (e.g., neglect) exert differential effects on neurodevelopment via distinct mechanisms (McLaughlin, Sheridan, & Lambert, 2014) and that even when threat and deprivation co‐occur, they work through dimension‐specific pathways to increase risk for psychopathology (Lambert, King, Monahan, & McLaughlin, 2017; Machlin, Miller, Snyder, McLaughlin, & Sheridan, 2019). Previous DMAP‐informed research largely relied on relatively small cross‐sectional samples focused on early childhood, adolescence, or young adulthood (Lambert et al., 2017; Machlin et al., 2019; Sosnowski et al., 2023). Thus, results might not be as generalisable to the rest of the population. Studying longitudinal cohort studies that follow children throughout similar developmental periods would facilitate the identification of naturally occurring dimensions of adversity in the wider population context.

Nevertheless, determining the dimensions of adversity has proven challenging. It is important to clarify that dimensional models can be theoretically driven (i.e., informed by frameworks such as DMAP) or empirically driven (i.e., informed by variable‐centred or person‐centred statistical methods; Lacey & Minnis, 2020). Theoretically driven studies informed by DMAP have applied confirmatory factor analysis (CFA) to validate two dimensions of threat and deprivation (Awada, Shelleby, Alfonso, & Keane, 2023; Miller, Machlin, McLaughlin, & Sheridan, 2021; Ning, Gondek, Pereira, & Lacey, 2023). However, confirmatory approaches such as CFA rely on a priori categorisations of ACEs, which can differ across research and practice even when using the DMAP framework. For example, in a study where mental health clinicians were first instructed to read the DMAP definitions and then categorise ACEs as either threat or deprivation according to their professional opinions, the majority categorised emotional abuse as deprivation (Henry et al., 2021), contrary to previous DMAP research that categorised emotional abuse as threat (Lambert et al., 2017; Miller et al., 2021). Furthermore, subjective practices in defining ACE measures can lead to inconsistent dimensions of adversity across studies (Wang et al., 2023), even if samples were drawn from the same dataset. Two independent studies that applied CFA on the same US birth cohort derived different numbers of dimensions, despite drawing from the same population and being informed by DMAP (Awada et al., 2023; Sisitsky et al., 2023). This was likely because each study chose different ACEs for each dimension and different measures for the same construct. For instance, Awada et al. (2023) derived two dimensions of threat and deprivation, which included parenting, financial, and neighbourhood measures of deprivation. However, Sisitsky et al. (2023) selected physical, emotional, and cognitive measures of deprivation and found that a four‐dimensional model of home threat, community threat, neglect, and lack of stimulation demonstrated better fit in their study. Altogether, these findings suggest that utilising a data‐driven approach to explore whether each measure might load onto different factors would help distinguish dimensions without being influenced by a priori categorisations.

Contrary to CFA, exploratory factor analysis (EFA) explores the underlying dimensions of interrelated measures without specifying any predefined structure. Empirically driven studies have used EFA as a variable‐centred method to derive varying dimensions, such as child maltreatment and household dysfunction, from the original 10 ACEs (Mersky et al., 2017). When using an expanded set of ACEs, EFA studies have identified 4–10 dimensions ranging from subtypes of threat and deprivation to carer psychopathology, socioeconomic disadvantage, and trauma exposure (Brieant et al., 2023; Mersky et al., 2017; Orendain, Anderson, Galván, Bookheimer, & Chung, 2023; Sosnowski et al., 2023). The variability in dimensions of adversity is likely in part due to sample‐specific differences in sociodemographic factors, developmental periods, and sample size. Thus, replicating a consistent protocol across two different populations could help distinguish if there are meaningful dimensions of adversity beyond sample‐specific artefacts.

Finally, it is also essential that researchers conceptualise their measures transparently to improve the reproducibility of research on ACEs, and this can be achieved through open science practices such as preregistration and openly shared code. We preregistered a data‐driven exploratory analysis of two longitudinal cohorts: the UK Millennium Cohort Study and the US Adolescent Brain Cognitive Development Study. Using empirically justified measures of ACEs, we aimed to (a) test if there were meaningful dimensions of ACEs across two populations and (b) investigate whether these dimensions were differentially associated with psychopathology in adolescence.

## Method

This study was preregistered and conforms to the STROBE guidelines for cohort studies (Appendix S1). We note that although we preregistered competing hypotheses about potential dimensions of ACEs that could emerge, our study aimed to be exploratory; details of our rationale can be found in our preregistration (https://osf.io/xqy9c). We hypothesised that (1a) ACEs would cluster as one higher‐order latent factor (supporting the cumulative risk score approach) or (1b) ACEs would cluster as two latent factors of threat and deprivation (supporting the dimensional approach of DMAP). We also hypothesised that (2a) ACEs conceptualised as the dimension of threat (e.g., abuse, violence) would be more strongly associated with psychopathology outcomes than deprivation, or (2b) ACEs conceptualised as the dimension of deprivation (e.g., poverty, neglect) would be more strongly associated with psychopathology outcomes than threat.

### Participants

The Millennium Cohort Study (MCS) is an ongoing longitudinal cohort study following over 18,000 children born between 2000 and 2002 in the United Kingdom (England, Scotland, Wales and Northern Ireland). This study analysed data from 18,539 children (48.6% females) after imputation (minimum *N* in the complete sample = 6,502) from sweeps 1 to 7 (ages 9 months, 3, 5, 7, 11, 14, and 17 years). MCS data was accessed through the publicly available UK Data Service. The NHS Research Ethics Committee provided ethical approval for all sweeps.

The Adolescent Brain Cognitive Development (ABCD) Study is an ongoing longitudinal cohort study following over 11,000 children born between 2006 and 2008 across 21 sites in the United States. This study analysed data from 11,876 children (47.8% females) after imputation (minimum *N* in the complete sample = 5,660) from baseline to three waves of follow‐up (ages 9–10, 10–11, 11–12, and 12–13 years). ABCD data was accessed through the publicly available US National Institute of Mental Health Archive. The Institutional Review Board (IRB) at the University of California San Diego provided ethical approval for most ABCD sites, with remaining sites obtaining local IRB approval.

### Measures

We systematically searched through both cohorts' data dictionaries and selected ACE measures based on previous MCS studies (Adjei et al., 2021; Bevilacqua, Kelly, Heilmann, Priest, & Lacey, 2021; Ning et al., 2023), ABCD studies (Baldwin et al., 2023; Brieant et al., 2023), and DMAP studies (Lambert et al., 2017; Machlin et al., 2019; Miller et al., 2021). We included a broad range of ACEs informed by previous research, such as peer victimisation (Finkelhor et al., 2015; WHO, 2018). To ensure consistency across measures, ACEs were binarized, where a score of 1 represented exposure to the adversity at least once throughout childhood. Within each measure for each time point, ACEs were aggregated at the item level and then binarized as present if they surpassed a clinical or statistical cut‐off. Cut‐offs were conservative to represent more severe exposure and were based on validated cut‐offs from previous studies (see Tables S1 and S2 for full details on how measures were derived). Where possible, we derived equivalent ACEs across both cohorts.

#### ACEs in MCS

We identified 173 items from the MCS and aggregated them across 9 months to age 14 years to create 18 composite ACE measures: poor parental mental health, frequent parental alcohol use, parental drug use, single parent, unhappy parental relationship, domestic violence, harsh parental discipline, parental smacking, negative home environment, peer victimisation, verbal victimisation, physical victimisation, theft victimisation, sexual victimisation, low cognitive stimulation, neighbourhood deprivation, unsafe home area, and low household income.

#### ACEs in ABCD

We identified 134 items from the ABCD Study and aggregated them across birth to age 11–12 years to create 18 composite ACE measures: parental psychopathology, parental alcohol abuse, parental drug abuse, parental separation, domestic violence, parental criminality, peer victimisation, cyber victimisation, physical abuse, emotional abuse, sexual abuse, emotional neglect, accident requiring medical attention, natural disaster, community violence, bereavement, unsafe neighbourhood, and low household income.

#### Psychopathology in MCS

Internalising and externalising symptoms were measured at age 17 using child self‐reports from the Strengths and Difficulties Questionnaire (SDQ), a 25‐item behavioural questionnaire with high reliability and validity (Goodman, 1997). We derived composite measures of internalising symptoms (emotional problems and peer problems) and externalising symptoms (conduct problems and hyperactivity/inattention) by summing and then standardising scores across the subscales.

#### Psychopathology in ABCD

Internalising and externalising symptoms were measured at age 12–13 using parent reports from the Child Behaviour Checklist (CBCL), a 119‐item behavioural questionnaire with excellent reliability and validity (Achenbach & Rescorla, 2001). We derived composite measures of internalising symptoms (anxious/depressed, withdrawn/depressed, and somatic complaints) and externalising symptoms (rule‐breaking, aggressive behaviour, and attention problems) by summing and then standardising scores across the subscales.

Scores from the SDQ and CBCL have been shown to be highly correlated and equally able to differentiate between high‐risk and low‐risk children (Goodman & Scott, 1999), ensuring outcome measures were broadly consistent across both cohorts.

### Statistical analysis

All analyses were conducted using R version 4.1.2 (http://www.r‐project.org/), and the code is available on GitHub.

#### Dimensions of ACEs

To identify the dimensions underlying ACEs, we conducted exploratory factor analysis (EFA) using the *psych* package (Revelle, 2015). We chose factor analysis instead of a person‐centred approach because the variable‐centred approach of factor analysis examines which ACEs cluster together regardless of population characteristics, whereas the person‐centred approach (e.g., latent class analysis) identifies subgroups of people who report similar ACE exposure in that particular population. We chose EFA over CFA as we aimed to explore the naturally occurring factor structure in both populations, although we did expect at least one threat‐related and one deprivation‐related factor based on previous research (McLaughlin et al., 2014). First, we conducted parallel analysis with 1,000 Monte Carlo simulations to determine the optimal number of factors to extract. EFA was conducted with an oblique rotation on the tetrachoric correlation matrix using weighted least squares estimation. We evaluated model fit using absolute and relative fit indices (RMSEA <0.06, RMSR <0.08, and TLI >0.95 indicated good fit; Hu & Bentler, 1999). We also considered which model had the “cleanest” factor structure, defined as factor loadings equal to or more than 0.30, with no or few item cross‐loadings (Costello & Osborne, 2005).

#### Associations between ACE dimensions and psychopathology

After selecting the best‐fitting model, we extracted continuous factor scores for each child, where higher scores reflected higher levels of the ACE dimension. We then ran univariate linear regressions to test the associations between each dimension and psychopathology outcomes. Next, we ran adjusted models including sex and ethnicity as covariates, given that previous research has suggested that sex and ethnicity are associated with both the clustering of ACEs and adolescent mental health (Jones, Pierce, & Shafer, 2022; Zhang & Monnat, 2022). Finally, we ran a multivariate multiple regression model including all dimensions, covariates, and psychopathology outcomes. As preregistered sensitivity analyses, we also tested for interactions between sex and ACE dimensions on psychopathology, given that some previous research found that sex moderated the relationships between ACEs and psychopathology (Houtepen, Heron, Suderman, Tilling, & Howe, 2018; Jones et al., 2022).

#### Missing data imputation

We imputed missing data with a random forest algorithm using the *missForest* package (Stekhoven & Buehlmann, 2012). We trained the random forest on the raw items comprising our exposure and outcome variables, covariates, and auxiliary variables. Auxiliary variables were sociodemographic indicators associated with missingness and ACEs (e.g., birthweight, smoking during pregnancy, home ownership at birth; Houtepen et al., 2018). For both the MCS and ABCD, we re‐derived the composite measures of ACEs and psychopathology from the imputed data and then replicated our analyses to ensure results were consistent across complete and imputed samples.

## Results

### Prevalence of ACEs in MCS and ABCD

Descriptive statistics are reported in Table 1, with information on missingness and sample attrition available online (Figures S1, S2, Tables S3 and S4). The most prevalent ACE in the MCS sample was ever having a single parent (51.30%) by age 14, whereas the least prevalent ACE was sexual victimisation (1.58%). The most prevalent ACE in the ABCD Study was parental psychopathology (39.93%), while the least prevalent ACE was physical abuse (1.03%). Levels of sexual victimisation in the MCS (1.58%) were approximately equivalent to sexual abuse in the ABCD (2.42%). 7.80% of MCS children were from households with domestic violence by age 14, while 9.68% of ABCD children had witnessed domestic violence by age 12.

**Table 1:** Descriptive statistics for the MCS and ABCD

Millennium Cohort Study (MCS)			
ACEs (9 months to 14 years)	Complete *n*	*n* Exposed	% Exposed
Poor parental mental health	18,312	3,721	20.07
Frequent parental alcohol use	18,521	2,385	12.86
Parental drug use	15,574	990	5.34
Single parent	18,521	9,510	51.30
Unhappy parental relationship	16,383	6,117	33.00
Domestic violence	16,233	1,446	7.80
Harsh parental discipline	15,163	7,276	39.25
Parental smacking	15,118	3,201	17.27
Negative home environment	13,863	675	3.64
Peer victimisation	16,420	4,477	24.15
Verbal victimisation	10,787	4,634	25.00
Physical victimisation	10,786	2,386	12.87
Theft victimisation	10,782	770	4.15
Sexual victimisation	10,781	293	1.58
Low cognitive stimulation	16,377	1,484	8.00
Neighbourhood deprivation	17,844	2,100	11.33
Unsafe home area	16,351	3,472	18.73
Low household income	18,513	7,586	40.92

Sociodemographic characteristics (baseline)	Complete *n*	%
Sex		
Male	9,526	51.38
Female	9,013	48.62
Ethnicity		
White	15,277	82.40
Black/Black British	666	3.59
Pakistani/Bangladeshi	1,265	6.82
Indian	465	2.51
Mixed	552	2.98
Other (inc. Chinese)	266	1.43
Maternal education		
Higher degree	635	3.44
First degree/diploma in higher educ.	3,813	20.65
A/AS/S levels	1,732	9.38
GCSE A*–C	6,167	33.40
GCSE D–G/no qualifications	5,600	30.33
Other (inc. overseas)	515	2.79

Psychopathology (17 years)	Complete *n*	*M* (*SD*)	Range
Internalising symptoms	9,398	5.62 (3.47)	0–20
Externalising symptoms	9,399	5.61 (3.29)	0–20

Adolescent Brain Cognitive Development (ABCD) Study			
ACEs (0–11/12 years)	Complete *n*	*n* exposed	% Exposed
Parental psychopathology	11,784	4,742	39.93
Parental alcohol abuse	11,876	1,720	14.48
Parental drug abuse	11,607	1,366	11.50
Parental separation	11,409	2,750	23.16
Domestic violence	11,836	1,150	9.68
Parental criminality	11,446	1,146	9.65
Peer victimisation	10,392	829	6.98
Cyber victimisation	10,361	930	7.83
Physical abuse	11,836	122	1.03
Emotional abuse	11,836	189	1.59
Sexual abuse	11,836	287	2.42
Emotional neglect	11,876	433	3.65
Accident requiring medical attention	11,836	1,347	11.34
Natural disaster	11,836	754	6.35
Community violence	11,836	187	1.57
Bereavement	11,836	3,876	32.64
Unsafe neighbourhood	11,441	2,056	17.31
Low household income	10,607	1,901	16.01

Sociodemographic characteristics (baseline)	Complete *n*	%
Sex		
Male	6,196	52.17
Female	5,680	47.83
Ethnicity		
White	7,522	63.34
Black/African American	2,269	19.11
Other race	800	6.74
Asian	716	6.03
American Indian/Alaska Native	346	2.91
Native Hawaiian/Pacific Islander	50	0.42
Parental education		
Postgraduate degree	4,043	34.04
Bachelor's degree	3,015	25.39
Some college degree	3,079	25.93
HS Diploma/GED	1,131	9.52
<HS Diploma	592	4.98

Psychopathology (12/13 years)	Complete *n*	*M* (*SD*)	Range
Internalising symptoms	6,169	5.13 (5.78)	0–44
Externalising symptoms	6,169	6.69 (7.89)	0–64

### Dimensions of adverse childhood experiences

Our data‐driven exploratory factor analysis revealed the four‐factor model as the best‐fitting according to model fit indices in the MCS (RMSEA = 0.09, RMSR = 0.03, TLI = 0.80) and ABCD (RMSEA = 0.09, RMSR = 0.04, TLI = 0.79). Three of the four factors emerged as consistent dimensions across both populations: parental threat, deprivation, and victimisation. Full details of how the four‐factor model holistically met the criteria as the best‐fitting model are available online (Appendices S2–S5, Figures S3–S17, Tables S5–S8).

In the MCS, the one‐factor model (Figure S5) indicated poor fit indices compared to the rest of the models (Table S5), and two ACE measures (low cognitive stimulation and sexual victimisation) had low loadings of 0.20. Parental smacking, frequent parental alcohol use, harsh parental discipline, and unhappy parental relationships did not load onto the one‐factor model. The two‐factor model (Figure S6) had better fit indices than the one‐factor model. Harsh parental discipline, parental smacking, and unhappy parental relationships did not load onto the two‐factor model. Although there appeared to be two factors of threat/deprivation‐related events and victimisation, there was no distinction between threat and deprivation ACEs to support DMAP. The three‐factor model (Figure S7) had better fit indices than the two‐factor model, with all items loading onto three factors. The second and third factors were moderately correlated (*r =* .30), but their respective ACE measures loaded onto distinct dimensions. The four‐factor model (Figure S8) was the second‐best fitting model in terms of fit indices, with all loadings equal to or above .30. The first three factors were moderately correlated with each other (*r =* .20), but their respective ACE measures loaded onto distinct dimensions. The five‐factor model (Figure S9) demonstrated the best fit indices, but the third factor consisted of only one item (parental drug use), indicating model instability. All five factors were correlated with each other (*r* = .20–.30). Overall consideration of the fit indices and factor structure suggested the four‐factor model fit the MCS data optimally, and these findings were replicated in the imputed sample (Appendix S3).

**Figure 1:**
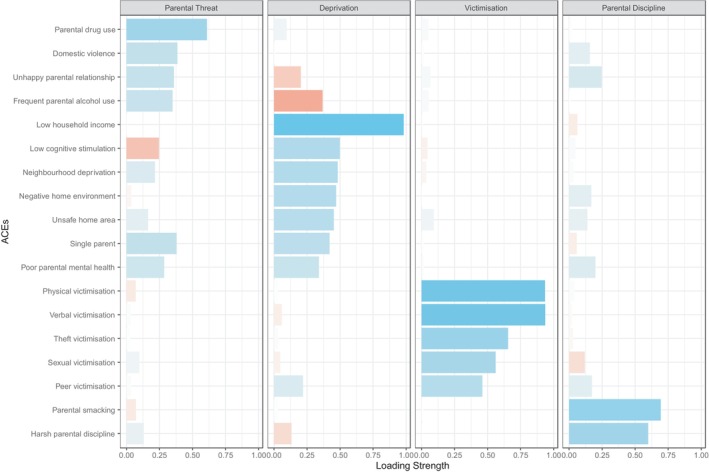
MCS factor loadings onto each dimension. Blue bars represent positive loadings of ACEs onto each dimension, whilst red bars represent negative loadings.

In the MCS, ACEs loaded onto four factors of parental threat, deprivation, victimisation, and parental discipline (Figure 1). The parental threat dimension included parental drug use, domestic violence, unhappy parental relationships, and frequent parental alcohol use. The deprivation dimension included low household income, low cognitive stimulation, neighbourhood deprivation, negative home environment, unsafe home area, single parent, and poor parental mental health. The victimisation dimension consisted of physical, verbal, theft, sexual, and peer victimisation. The parental discipline dimension consisted of parental smacking and harsh parental discipline.

In the ABCD, the one‐factor model (Figure S13) indicated poor fit indices compared to the rest of the models (Table S7), and one ACE measure (peer victimisation) had a low loading of 0.20. Emotional neglect did not load onto the one‐factor model. The two‐factor model (Figure S14) had better fit indices than the one‐factor model. Peer victimisation did not load onto the two‐factor model. The first and second factors were correlated (*r =* .50), but their respective ACE measures loaded onto distinct dimensions. Although there appeared to be two factors of threat/deprivation‐related events and traumatic events, there was no distinction between threat and deprivation ACEs to support DMAP. The three‐factor model (Figure S15) had better fit indices than the two‐factor model, with all items loading onto three factors. The first and second factors were identical to the two‐factor model, except that emotional neglect, peer victimisation and cyber victimisation loaded onto the third factor. The four‐factor model (Figure S16) demonstrated the best fit indices, with all loadings equal to or above .30. The first three factors were moderately correlated with each other (*r =* .30–.40), but their respective ACE measures loaded onto distinct dimensions. Overall consideration of the fit indices and factor structure criteria suggested the four‐factor model fit the ABCD data optimally, and these findings were replicated in the imputed sample (Appendix S5).

**Figure 2:**
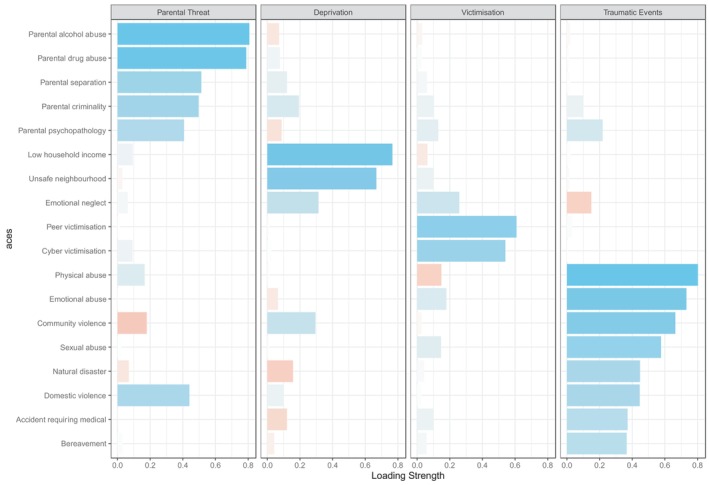
ABCD factor loadings onto each dimension. Blue bars represent positive loadings of ACEs onto each dimension, whilst red bars represent negative loadings.

In the ABCD, ACEs loaded onto four factors of parental threat, deprivation, victimisation, and traumatic events (Figure 2). The parental threat dimension included parental alcohol abuse, parental drug abuse, parental separation, parental criminality, and parental psychopathology. The deprivation dimension included low household income, unsafe neighbourhoods, and emotional neglect. The victimisation dimension consisted of peer and cyber victimisation. The traumatic events dimension consisted of physical abuse, emotional abuse, community violence, sexual abuse, natural disaster, domestic violence, accident requiring medical attention, and bereavement.

### Associations between adversity dimensions and adolescent psychopathology

Within both cohorts, dimensions of adversity were differentially associated with adolescent psychopathology. In the MCS, univariate analyses revealed that parental threat was associated with internalising symptoms (β = .17, 95% CI 0.16–0.18, *p* < .001), as well as externalising symptoms (β = .21, 95% CI 0.20–0.23, *p* < .001). These associations remained after adjusting for covariates (Tables 2 and 3). There was no significant association between deprivation and internalising symptoms (β = −.01, 95% CI –0.03 to 0.00004, *p* = .051) or externalising symptoms (β = −.003, 95% CI –0.02 to 0.01, *p* = .661). After adjusting for sex and race, deprivation remained unassociated with internalising and externalising symptoms (Tables 2 and 3). Victimisation was associated with internalising symptoms (β = .37, 95% CI 0.36–0.39, *p* < .001) and externalising symptoms (β = .38, 95% CI 0.37–0.40, *p* < .001) and these associations remained after adjusting for covariates (Tables 2 and 3). Parental discipline showed a small association with internalising symptoms (β = .16, 95% CI 0.15–0.18, *p* < .001), and a stronger association with externalising symptoms (β = .31, 95% CI 0.29–0.32, *p* < .001). After covariate adjustment, parental discipline remained associated with both internalising and externalising symptoms (Tables 2 and 3). In the multivariate adjusted model, victimisation appeared to be the most strongly associated with internalising and externalising symptoms, followed by parental discipline and parental threat (Tables 2 and 3). Deprivation remained unassociated with psychopathology after accounting for the other three dimensions.

**Table 2:** MCS and ABCD adjusted associations between ACE dimensions and internalising symptoms

ACE dimension	Internalising symptoms	Multivariate model
	β (95% CI)	β (95% CI)
*Millennium Cohort Study (MCS)*		
Parental threat	.17*** (0.15, 0.18)	.06*** (0.05, 0.08)
Deprivation	−.002 (−0.02, 0.01)	.003 (−0.01, 0.02)
Victimisation	.38*** (0.36, 0.39)	.34*** (0.33, 0.36)
Parental discipline	.18*** (0.16, 0.19)	.08*** (0.07, 0.10)
*Adolescent Brain Cognitive Development (ABCD) Study*		
Parental threat	.10*** (0.08, 0.12)	.07*** (0.05, 0.09)
Deprivation	−.01 (−0.03, 0.01)	−.05*** (−0.07, −0.03)
Victimisation	.13*** (0.11, 0.15)	.11*** (0.10, 0.13)
Traumatic events	.08*** (0.07, 0.10)	.05*** (0.03, 0.07)

All regression models were adjusted for sex and ethnicity. ACE dimensions and internalising symptoms were standardised. β = standardised regression coefficient; CI, confidence interval.

*p < .05; **p < .01; ***p < .001.

**Table 3:** MCS and ABCD adjusted associations between ACE dimensions and externalising symptoms

ACE dimension	Externalising symptoms	Multivariate model
	β (95% CI)	β (95% CI)
*Millennium Cohort Study (MCS)*		
Parental threat	.22*** (0.20, 0.23)	.08*** (0.07, 0.10)
Deprivation	.01 (−0.01, 0.02)	.01 (−0.002, 0.03)
Victimisation	.38*** (0.37, 0.39)	.31*** (0.30, 0.33)
Parental discipline	.30*** (0.29, 0.32)	.21*** (0.20, 0.23)
*Adolescent Brain Cognitive Development (ABCD) Study*		
Parental threat	.11*** (0.10, 0.13)	.08*** (0.06, 0.10)
Deprivation	.02 (−0.001, 0.04)	−.02** (−0.04, −0.004)
Victimisation	.15*** (0.13, 0.17)	.13*** (0.11, 0.15)
Traumatic events	.09*** (0.07, 0.11)	.04*** (0.02, 0.06)

All regression models were adjusted for sex and ethnicity. ACE dimensions and externalising symptoms were standardised. β, standardised regression coefficient; CI, confidence interval.

*p < .05; **p < .01; ***p < .001.

In the ABCD, univariate analyses revealed that parental threat was also associated with internalising symptoms (β = .09, 95% CI 0.07–0.11, *p* < .001) and externalising symptoms (β = .11, 95% CI 0.09–0.13, *p* < .001) and remained associated after covariate adjustment (Tables 2 and 3). Deprivation was not associated with externalising symptoms (β = −.004, 95% CI –0.02 to 0.01, *p* = .633). While a very small negative association with internalising symptoms was found in univariate analyses (β = −.05, 95% CI –0.07 to −0.03, *p* < .001), this was not the case after adjusting for sex and race (Tables 2 and 3). Victimisation was associated with internalising symptoms (β = .13, 95% CI 0.11–0.15, *p* < .001) and externalising symptoms (β = .15, 95% CI 0.13–0.17, *p* < .001) and remained associated with these outcomes after adjusting for covariates (Tables 2 and 3). There were small associations between traumatic events and internalising symptoms (β = .07, 95% CI 0.06–0.09, *p* < .001), as well as externalising symptoms (β = .09, 95% CI 0.07–0.11, *p* < .001). Traumatic events remained associated with psychopathology after adjusting for covariates (Tables 2 and 3). Similar to the MCS, in the multivariate adjusted model, victimisation was the most strongly associated with internalising and externalising symptoms, followed by parental threat and traumatic events (Tables 2 and 3). After accounting for the other dimensions, deprivation had very small negative associations with internalising symptoms (β = −.05, 95% CI –0.07 to −0.03, *p* < .001) and externalising symptoms (β = −.02, 95% CI –0.04 to −0.004, *p* < .001). However, these associations were extremely small and not found in the complete case sample (Appendix S8, Tables S13 and S14).

In preregistered sensitivity analyses, we also investigated whether sex interacted with adversity to influence psychopathology. We found small, statistically significant interactions between sex and specific adversity dimensions; specifically, girls in the MCS who experienced victimisation were at slightly higher risk for internalising symptoms than boys, while boys in the ABCD who experienced parental threat were at slightly higher risk for externalising symptoms than girls (Appendices S7 and S9). However, these results were not replicated across cohorts, and we did not find sex‐by‐adversity interactions for the majority of adversity dimensions and psychopathology outcomes.

Associations between adversity dimensions and psychopathology were broadly consistent across the complete and imputed samples, with detailed results available online (Appendices S6, S8, Tables S9, S10, Tables S13 and S14). For both populations, we established that multicollinearity between ACE dimensions was very unlikely due to low variance inflation factors (<1.30), high tolerance values (>0.80), and dimension intercorrelations (mean *r* = .17) (Tables S11, S12, Tables S15 and S16).

## Discussion

To our knowledge, this is the largest preregistered data‐driven analysis of adversity dimensions using two contemporary longitudinal cohorts from the UK and US. We identified four dimensions of parental threat, deprivation, victimisation, and parental discipline in the MCS, and four dimensions of parental threat, deprivation, victimisation, and traumatic events in the ABCD. The parental threat and deprivation dimensions partially support our hypothesis that ACEs would cluster as threat and deprivation. Three of the four dimensions of adversity emerged consistently across both populations: parental threat, deprivation, and victimisation. The consistency of these dimensions is striking given the sociodemographic differences between the UK and US populations. This suggests that these dimensions are meaningful and not sample‐specific, as they were identified despite the different instruments and informants used. In both populations, low household income and lack of neighbourhood safety loaded onto the deprivation dimension, peer victimisation clustered with other forms of interpersonal victimisation, and parental drug and alcohol use clustered with other parental threat‐related ACEs. Of note, our dimensions of parental threat and deprivation converged with equivalent dimensions from recent ABCD studies that also applied EFA, demonstrating the advantage of exploratory data‐driven methods (Brieant et al., 2023; Orendain et al., 2023).

Two dimensions of childhood adversity were unique to each cohort: parental discipline in the MCS and traumatic events in the ABCD. In the MCS, the parental discipline dimension comprised parental smacking and harsh parental discipline. Contrary to a recent MCS study that constructed the threat dimension a priori from interparental violence and parental discipline in a CFA (Ning et al., 2023), our data‐driven EFA demonstrated that parental discipline emerged as a distinct dimension from parental threat. In the ABCD, the traumatic events dimension included ACEs such as physical, emotional, and sexual abuse but also bereavement and natural disasters. The traumatic events dimension in our study was identical to the trauma exposure dimension identified by a recent EFA of cross‐sectional data from the ABCD (Brieant et al., 2023). It appears that the parental discipline and traumatic events dimensions did not replicate across populations because they each consisted of ACEs from measuring instruments specific to each cohort, i.e., the MCS parental discipline dimension from the Conflict Tactics Scale (Straus, 1979) and the ABCD traumatic events dimension from the Kiddie Schedule for Affective Disorders and Schizophrenia (K‐SADS; Kaufman et al., 1997). The convergence of these instrument‐specific dimensions highlights how data‐driven dimensions of adversity might be partly driven by shared method variance. For researchers analysing secondary datasets, dimensions of adversity are ultimately constrained by the measuring instruments used, which vary considerably across studies (e.g., differences in adversity type measured, informant, timing, and phrasing of questions to measure the same construct). These cumulative differences present a sizeable challenge in conceptualising a universal model for adversity that transcends divergent measures across studies and likely account for the discrepant dimensions identified across cohorts (e.g., parental discipline in MCS and traumatic events in ABCD) despite the three common dimensions.

We found that dimensions of adversity in the MCS and ABCD Study were associated with adolescent psychopathology in distinct ways. Parental threat was consistently associated with internalising and externalising symptoms across both cohorts, in line with previous DMAP‐informed research (Awada et al., 2023; Miller et al., 2021; Sosnowski et al., 2023). However, there was no association between deprivation and adolescent psychopathology in the MCS, and there were very small associations between deprivation and adolescent psychopathology in the ABCD. These findings support our hypothesis that the dimension of threat would be more strongly associated with psychopathology than deprivation. Similarly, recent ABCD studies that conceptualised deprivation as a dimension of socioeconomic disadvantage or scarcity did not find associations with internalising and externalising symptoms (Brieant et al., 2023) or only with internalising symptoms (Orendain et al., 2023). Whilst it seems that deprivation does emerge as a meaningful dimension, its impact on adolescent psychopathology appears to be inconsistent in these samples, and this is likely due to the complexity of measuring deprivation as a multidimensional construct. Research has shown that the strength and consistency of the association between deprivation and psychopathology vary according to the dimension of deprivation used (Díaz, Hessel, Avendano, & Evans‐Lacko, 2022; Lund et al., 2010). For example, there is evidence for individual deprivation (e.g., educational non‐attendance) being more strongly associated with adolescent psychopathology than material deprivation (e.g., household overcrowding) (Díaz et al., 2022), as well as more consistent associations found between subjective social status and adolescent mental disorders compared to objective socioeconomic indicators (McLaughlin, Costello, Leblanc, Sampson, & Kessler, 2012). As the deprivation dimensions we derived in the MCS and ABCD cohorts included a broad range of measures of deprivation, this might have contributed to the inconsistent associations with adolescent psychopathology. Thus, future research could clarify which dimensions of deprivation influence adolescent psychopathology within the context of co‐occurring ACEs.

Notably, we found stable associations between victimisation and internalising and externalising symptoms, which displayed the strongest effect sizes over and above the other ACE dimensions for both populations. In the MCS, associations were particularly strong and might have been inflated by shared method variance, as victimisation ACEs and adolescent psychopathology were self‐reported. However, shared method variance cannot completely explain the associations in the ABCD, as adolescent psychopathology was parent‐reported. Moreover, the ABCD victimisation dimension consisted of only peer and cyber victimisation, yet victimisation persisted in being more strongly associated with adolescent psychopathology compared to the traumatic events dimension, which consisted of maltreatment‐related ACEs such as physical, emotional, and sexual abuse. Our findings align with quasi‐experimental meta‐analytic evidence suggesting a causal relationship between bullying victimisation and mental health problems (Schoeler, Duncan, Cecil, Ploubidis, & Pingault, 2018) and suggest that when conceptualising ACEs as dimensions, future research should investigate whether victimisation ACEs impact adolescent psychopathology via different mechanisms than other dimensions (e.g., threat and deprivation). For example, the interpersonal nature of victimisation, particularly when the perpetrator is a peer, might impair adolescent mental health in a targeted way unlike other less relational ACEs. Importantly, we recommend the inclusion of peer victimisation as an ACE in future studies, as many recent studies that derived adversity dimensions did not include peer victimisation (e.g., Awada et al., 2023; Brieant et al., 2023; Lambert et al., 2017; Machlin et al., 2019; Miller et al., 2021; Ning et al., 2023; Orendain et al., 2023; Sosnowski et al., 2023).

Regarding the unique dimensions within each cohort, parental discipline and traumatic events demonstrated small, consistent associations with adolescent psychopathology. In the MCS, parental discipline showed larger associations with externalising symptoms compared to internalising symptoms, supporting previous longitudinal evidence for the reciprocal relationship between parents' use of harsh discipline and children's externalising behaviour (Lansford et al., 2011). In the ABCD, traumatic events showed stable associations with both internalising and externalising symptoms. As this relationship has so far been demonstrated by cross‐sectional evidence from the ABCD (Brieant et al., 2023; Orendain et al., 2023), our longitudinal findings extend the literature by reducing the possibility of reverse causality.

Our findings should be interpreted in the context of several limitations. First, most of our ACE measures were parent‐reported, which might lead to underreporting due to social desirability bias. Second, although we attempted to derive equivalent ACEs across both populations, we were limited by the available measures, which tended to vary in severity across cohorts. We endeavoured to minimise these differences by applying conservative cut‐offs and clarifying if the ACE measure was relative or absolute. Third, both longitudinal cohorts are affected by selective attrition, with less affluent families and more marginalised groups being more likely to drop out of both cohorts (Connelly & Platt, 2014; Feldstein Ewing et al., 2022). However, we imputed missing data with a random forest algorithm trained on sociodemographic variables associated with missingness to mitigate this selection bias. Fourth, we note that our dimension labels are broad categorisations. We acknowledge the duality in the nature of some adversities (e.g., parent psychopathology could be labelled as threat or deprivation, as indicated by cross‐loadings between factors), and this highlights a broader limitation of DMAP: there is no clear delineation of either threat or deprivation experiences due to the duality and frequent co‐occurrence of ACEs. Lastly, we cannot infer that different dimensions of ACEs caused internalising and externalising symptoms in adolescence, as there might have been unmeasured confounding (e.g., from genetic influences; Baldwin et al., 2023).

Nevertheless, our findings advance the literature with implications for how we should best conceptualise ACEs. We provide empirical support for the utility of the dimensional approach of conceptualising ACEs, which may have advantages over the cumulative risk approach, by showcasing how different dimensions of ACEs were differentially associated with adolescent psychopathology. We demonstrated that applying data‐driven exploratory factor analysis without a priori categorisations successfully captured common underlying dimensions of parental threat, deprivation, and victimisation across two distinct populations. These dimensions are meaningful as they converged despite the sociodemographic and measurement differences between the UK MCS and US ABCD Study. Whilst our dimensions of parental threat and deprivation provide partial support for DMAP, our findings also suggest that existing conceptual frameworks of ACEs should be expanded to include victimisation as a distinct dimension, as victimisation demonstrated the strongest associations with adolescent psychopathology over and above the other dimensions. This offers potential avenues for future research regarding mechanisms, as ACEs within the victimisation dimension might impact adolescent psychopathology via different pathways than threat and deprivation. Finally, we provide an open science resource of ACE measures in the MCS and ABCD, many of which have not been derived before. Our code can be replicated by future researchers and will hopefully contribute to facilitating the reproducibility of ACE‐related research in both the MCS and ABCD datasets.

**Key points**

- There is not yet a consensus on the best way to conceptualise adverse childhood experiences (ACEs).
- By applying data‐driven analyses and consistent practices in defining ACE measures, our study derived meaningful dimensions of adversity across two distinct populations, the UK Millennium Cohort Study (MCS) and the US Adolescent Brain Cognitive Development (ABCD) Study.
- Our findings expand dimensional theories of childhood adversity by suggesting that in addition to threat and deprivation, victimisation is a distinct dimension of adversity that has the strongest associations with adolescent psychopathology.
- To facilitate reproducibility in future research, we provide an open science resource of ACE measures in the MCS and ABCD, many of which have not been derived before.

## Supporting information


**Appendix S1.** STROBE (Strengthening the Reporting of Observational Studies in Epidemiology) Checklist of items that should be included in reports of cohort studies.
**Appendix S2.** Exploratory factor analysis (EFA) on the MCS complete sample.
**Appendix S3.** Exploratory factor analysis (EFA) on the MCS imputed sample.
**Appendix S4.** Exploratory factor analysis (EFA) on the ABCD complete sample.
**Appendix S5.** Exploratory factor analysis (EFA) on the ABCD imputed sample.
**Appendix S6.** Associations between adversity dimensions and adolescent psychopathology in the MCS complete sample.
**Appendix S7.** Gender interactions in the MCS complete sample.
**Appendix S8.** Associations between adversity dimensions and adolescent psychopathology in the ABCD complete sample.
**Appendix S9.** Gender interactions in the ABCD complete sample.
**Table S1.** Criteria for deriving exposure and outcome measures from the Millennium Cohort Study.
**Table S2.** Criteria for deriving exposure and outcome measures from the Adolescent Brain Cognitive Development Study.
**Table S3.** Missing data per variable before multiple imputation in the MCS.
**Table S4.** Missing data per variable before multiple imputation in the ABCD Study.
**Table S5.** EFA fit indices for one‐ through five‐factor models for the MCS complete sample.
**Table S6.** EFA fit indices for one‐ through five‐factor models for the MCS imputed sample.
**Table S7.** EFA fit indices for one‐ through four‐factor models for the ABCD complete sample.
**Table S8.** EFA fit indices for one‐ through four‐factor models for the ABCD imputed sample.
**Table S9.** Adjusted associations between ACE dimensions and internalising symptoms in the MCS complete sample.
**Table S10.** Adjusted associations between ACE dimensions and externalising symptoms in the MCS complete sample.
**Table S11.** Variance inflation factor (VIF) for the multivariate model in the MCS complete sample.
**Table S12.** Variance inflation factor (VIF) for the multivariate model in the MCS imputed sample.
**Table S13.** Adjusted associations between ACE dimensions and internalising symptoms in the ABCD complete sample.
**Table S14.** Adjusted associations between ACE dimensions and externalising symptoms in the ABCD complete sample.
**Table S15.** Variance inflation factor (VIF) and tolerance values for the multivariate model for the ABCD complete sample.
**Table S16.** Variance inflation factor (VIF) and tolerance values for the multivariate model for the ABCD imputed sample.
**Figure S1.** Flowchart of participants in the Millennium Cohort Study.
**Figure S2.** Flowchart of participants in the ABCD Study.
**Figure S3.** Correlation matrix depicting tetrachoric correlations between ACEs in the MCS complete sample.
**Figure S4.** Parallel analysis scree plot generated after 1,000 simulations on the MCS complete sample.
**Figure S5.** One‐factor model for the MCS complete sample.
**Figure S6.** Two‐factor model for the MCS complete sample.
**Figure S7.** Three‐factor model for the MCS complete sample.
**Figure S8.** Four‐factor model for the MCS complete sample.
**Figure S9.** Five‐factor model for the MCS complete sample.
**Figure S10.** Four‐factor model for the MCS imputed sample.
**Figure S11.** Correlation matrix depicting tetrachoric correlations between ACEs in the ABCD complete sample.
**Figure S12.** Parallel analysis scree plot generated after 1,000 simulations on the ABCD complete sample.
**Figure S13.** One‐factor model for the ABCD complete sample.
**Figure S14.** Two‐factor model for the ABCD complete sample.
**Figure S15.** Three‐factor model for the ABCD complete sample.
**Figure S16.** Four‐factor model for the ABCD complete sample.
**Figure S17.** Four‐factor model for the ABCD imputed sample.

## Data Availability

The Millennium Cohort Study (MCS) data is publicly available through the UK Data Service, a national digital repository and data sharing platform based at the University of Essex. Information on how to access MCS data through the UK Data Service is available on the CLS data‐sharing webpage: https://cls.ucl.ac.uk/data‐access‐training/data‐access/accessing‐data‐via‐public‐repositories/. Instructions on how to submit a data request to the CLS Data Access Committee are available at https://cls.ucl.ac.uk/data‐access‐training/data‐access/accessing‐data‐directly‐from‐cls/. The Adolescent Brain Cognitive Development (ABCD) Study data, including all assessment domains, are released annually to the research community. Information on how to access ABCD data through the National Institute of Mental Health Data Archive (NDA) is available on the ABCD Study data‐sharing webpage: https://abcdstudy.org/scientists_data_sharing.html. Instructions on how to create an NDA study are available at https://nda.nih.gov/training/modules/study.html. The ABCD data repository grows and changes over time.
