# Supplementary material for: Do meaningful dimensions of childhood adversity exist? Data‐driven evidence from two prospective cohort studies
Source: J Child Psychol Psychiatry. 2024 Dec 17;66(6):868–80. doi: 10.1111/jcpp.14098 (PMC12062845; doi:10.1111/jcpp.14098)
Supplement: Supplementary file 1 — Appendix S1. STROBE (Strengthening the Reporting of Observational Studies in Epidemiology) Checklist of items that should be included in reports of cohort studies. Appendix S2. Exploratory factor analysis (EFA) on the MCS complete sample. Appendix S3. Exploratory factor analysis (EFA) on the MCS imputed sample. Appendix S4. Exploratory factor analysis (EFA) on the ABCD complete sample. Appendix S5. Exploratory factor analysis (EFA) on the ABCD imputed sample. Appendix S6. Associations between adversity dimensions and adolescent psychopathology in the MCS complete sample. Appendix S7. Gender interactions in the MCS complete sample. Appendix S8. Associations between adversity dimensions and adolescent psychopathology in the ABCD complete sample. Appendix S9. Gender interactions in the ABCD complete sample. Table S1. Criteria for deriving exposure and outcome measures from the Millennium Cohort Study. Table S2. Criteria for deriving exposure and outcome measures from the Adolescent Brain Cognitive Development Study. Table S3. Missing data per variable before multiple imputation in the MCS. Table S4. Missing data per variable before multiple imputation in the ABCD Study. Table S5. EFA fit indices for one‐ through five‐factor models for the MCS complete sample. Table S6. EFA fit indices for one‐ through five‐factor models for the MCS imputed sample. Table S7. EFA fit indices for one‐ through four‐factor models for the ABCD complete sample. Table S8. EFA fit indices for one‐ through four‐factor models for the ABCD imputed sample. Table S9. Adjusted associations between ACE dimensions and internalising symptoms in the MCS complete sample. Table S10. Adjusted associations between ACE dimensions and externalising symptoms in the MCS complete sample. Table S11. Variance inflation factor (VIF) for the multivariate model in the MCS complete sample. Table S12. Variance inflation factor (VIF) for the multivariate model in the MCS imputed sample. Table S13. Adjusted associations b [file JCPP-66-868-s001.docx]

**Do meaningful dimensions of childhood adversity exist?**

**Data-driven evidence from two prospective cohort studies.**

*Supporting Information*

Athena R.W. Chow^1^, BSc; Jessie R. Baldwin, PhD^2,3^; Lucy Bowes^1^, PhD

**Authors’ affiliations**

^1^ Department of Experimental Psychology, Medical Sciences Division, University of Oxford, Oxford, UK.

^2^ Department of Clinical, Educational and Health Psychology, Division of Psychology and Language Sciences, University College London, London, UK.

^3^ Social, Genetic and Developmental Psychiatry Centre, Institute of Psychiatry, Psychology and Neuroscience, King’s College London, London, UK.

# Appendix S1. STROBE (Strengthening the Reporting of Observational studies in Epidemiology) Checklist of items that should be included in reports of cohort studies.

|  | Item | Recommendation | Page |
| --- | --- | --- | --- |
| **Title and abstract** | 1 | (*a*) Indicate the study’s design with a commonly used term in the title or the abstract | 1-2 |
|  |  | (*b*) Provide in the abstract an informative and balanced summary of what was done and what was found | 2 |
| Introduction | | |  |
| Background/rationale | 2 | Explain the scientific background and rationale for the investigation being reported | 3-6 |
| Objectives | 3 | State specific objectives, including any prespecified hypotheses | 6 |
| Methods | | |  |
| Study design | 4 | Present key elements of study design early in the paper | 7-9 |
| Setting | 5 | Describe the setting, locations, and relevant dates, including periods of recruitment, exposure, follow-up, and data collection | 7 |
| Participants | 6 | (*a*) Give the eligibility criteria, and the sources and methods of selection of participants. Describe methods of follow-up | 7 |
|  |  | (*b*) For matched studies, give matching criteria and number of exposed and unexposed | N/A |
| Variables | 7 | Clearly define all outcomes, exposures, predictors, potential confounders, and effect modifiers. Give diagnostic criteria, if applicable | 8-9 |
| Data sources/ measurement | 8 | For each variable of interest, give sources of data and details of methods of assessment (measurement). Describe comparability of assessment methods if there is more than one group | Tables S1-S2 |
| Bias | 9 | Describe any efforts to address potential sources of bias | 11 |
| Study size | 10 | Explain how the study size was arrived at | 7-10, Figures S1-S2, Tables S3-S4 |
| Quantitative variables | 11 | Explain how quantitative variables were handled in the analyses. If applicable, describe which groupings were chosen and why | 7-11, Tables S1-S2 |
| Statistical methods | 12 | (*a*) Describe all statistical methods, including those used to control for confounding | 10-11 |
|  |  | (*b*) Describe any methods used to examine subgroups and interactions | 10-11 |
|  |  | (*c*) Explain how missing data were addressed | 11 |
|  |  | (*d*) If applicable, explain how loss to follow-up was addressed | 11 |
|  |  | (*e*) Describe any sensitivity analyses | 16-17 |
| Results | | |  |
| Participants | 13 | (a) Report numbers of individuals at each stage of study—eg numbers potentially eligible, examined for eligibility, confirmed eligible, included in the study, completing follow-up, and analysed | Figures S1-S2 |
|  |  | (b) Give reasons for non-participation at each stage | Figures S1-S2 |
|  |  | (c) Consider use of a flow diagram | Figures S1-S2 |
| Descriptive data | 14 | (a) Give characteristics of study participants (eg demographic, clinical, social) and information on exposures and potential confounders | Table 1 |
|  |  | (b) Indicate number of participants with missing data for each variable of interest | Tables S3-S4 |
|  |  | (c) Summarise follow-up time (eg, average and total amount) | Figures S1-S2 |
| Outcome data | 15 | Report numbers of outcome events or summary measures over time | Figures S1-S2 |
| Main results | 16 | (*a*) Give unadjusted estimates and, if applicable, confounder-adjusted estimates and their precision (eg, 95% confidence interval). Make clear which confounders were adjusted for and why they were included | 15-17 |
|  |  | (*b*) Report category boundaries when continuous variables were categorized | Tables S1-S2 |
|  |  | (*c*) If relevant, consider translating estimates of relative risk into absolute risk for a meaningful time period | N/A |
| Other analyses | 17 | Report other analyses done—eg analyses of subgroups and interactions, and sensitivity analyses | S2-S9, Figures S3-S17, Tables S5-S16 |
| Discussion | | |  |
| Key results | 18 | Summarise key results with reference to study objectives | 17-22 |
| Limitations | 19 | Discuss limitations of the study, taking into account sources of potential bias or imprecision. Discuss both direction and magnitude of any potential bias | 21-22 |
| Interpretation | 20 | Give a cautious overall interpretation of results considering objectives, limitations, multiplicity of analyses, results from similar studies, and other relevant evidence | 17-22 |
| Generalisability | 21 | Discuss the generalisability (external validity) of the study results | 21-22 |
| Other information | | |  |
| Funding | 22 | Give the source of funding and the role of the funders for the present study and, if applicable, for the original study on which the present article is based | 23 |

*Note.* Page numbers refer to pages in the main text, while ‘S’ refers to items in this supplementary document. Information on the STROBE Initiative is available at http://www.strobe-statement.org.

**Table S1.** Criteria for deriving exposure and outcome measures from the Millennium Cohort Study.

| Measure | Informant | Assessment type | Exposure period | Assessment  phase | Criteria for coding measure as present |
| --- | --- | --- | --- | --- | --- |
| Poor parental mental health | Parent | Rutter Malaise Inventory (RMI);  Kessler (K6) Scale | 9 months, 3, 5, 7, 11, and 14 years | Sweeps 1-6 | At 9 months, the Rutter Malaise Inventory (RMI) scale was used to measure parental mental health in the last 30 days (e.g., “do you often feel miserable or depressed?”, “does your heart often race like mad?”). Scores were summed and then dichotomised (cut-off score ≥ 4; Adjei et al., 2022). At 3, 5, 7, 11, and 14 years, the Kessler (K6) scale was used to measure parental mental health in the last 30 days (e.g., “so depressed nothing could cheer you up”, “nervous”). Scores were summed and then dichotomised (cut-off score ≥ 13; Straatmann et al., 2020). If parents reported poor mental health for at least 1 out of 6 sweeps, poor parental mental health was coded as present. |
| Frequent parental alcohol use | Parent | Parent interview (alcohol consumption) | 9 months, 3, 5, 7, 11, and 14 years | Sweeps 1-6 | Parents reported the frequency of their alcohol use which was dichotomised (every day/5-6 times per week = 1 vs 3-4 times per week/1-2 times per week/less than once a month/never = 0; Straatmann et al., 2020). If parents reported frequent alcohol use for at least 1 out of 6 sweeps, frequent parental alcohol use was coded as present. |
| Parental drug use | Parent | Parent interview (substance abuse) | 3, 5, and 14 years | Sweeps 2, 3, 6 | Parents reported the frequency of their drug use, which was dichotomised (regularly/occasionally = 1 vs never = 0; Bevilacqua et al., 2020). If parents reported drug use for at least 1 out of 3 sweeps, parental drug use was coded as present. |
| Single parent | Parent | Parent interview (marital status) | 9 months, 3, 5, 7, 11, and 14 years | Sweeps 1-6 | Parents reported their marital status, which was dichotomised (divorced/legally separated/widowed/single = 1 vs married/remarried/civil partnership = 0). If parents reported a single parent status for at least 1 out of 6 sweeps, the single parent measure was coded as present. |
| Unhappy parental relationship | Parent | Golombok Rust Inventory of Marital State (GRIMS) | 9 months, 3, 5, 7, 11, and 14 years | Sweeps 1-6 | Parents reported how happy they were with their relationship with their partner on a 7-point scale (1 = very unhappy and 7 being very happy), which was dichotomised (very unhappy [1]/2/3 = 1 vs 4/5/6/very happy [7] = 0). If parents reported an unhappy relationship for at least 1 out of 6 sweeps, unhappy parental relationship was coded as present. |
| Domestic violence | Parent | Golombok Rust Inventory of Marital State (GRIMS) | 9 months, 3, 5, 7, 11, and 14 years | Sweeps 1-6 | Parents reported whether their partner had ever used force in their relationship, which was dichotomised (yes = 1 vs no = 0). If parents reported experiencing this for at least 1 out of 6 sweeps, domestic violence was coded as present. |
| Harsh parental discipline | Parent | Straus Conflict Tactics scale (CTS) | 3, 5, and 7 years | Sweeps 2-4 | The Straus Conflict Tactics scale was used to measure parental discipline practices (e.g., “tells child off when naughty”). Scores were summed and then dichotomised (with a cut-off score ≥ 5 based on previous research; Bevilacqua et al., 2021). If parents surpassed the cut-off for at least 1 out of 3 sweeps, harsh parental discipline was coded as present. |
| Parental smacking | Parent | Straus Conflict Tactics scale (CTS) | 3, 5, and 7 years | Sweeps 2-4 | Parents reported whether they smacked their child, which was dichotomised (daily/once a week or more/once a month = 1 vs never/rarely = 0; Bevilacqua et al., 2021). If parents smacked their child for at least 1 out of 3 sweeps, parental smacking was coded as present. |
| Negative home environment | Interviewer | Home Observation for Measurement of the Environment (HOME-SF) | 3 years | Sweep 2 | An interviewer visited the cohort member’s home to observe the physical environment (e.g., “child’s in-home play environment safe”) and responsiveness of the mother (e.g., “mother’s voice positive when speaking to child”). Scores were negatively coded, summed, and then dichotomised (scores that were 2 standard deviations above the mean were coded as a negative home environment; Totsika & Sylva, 2004). |
| Peer victimisation | Parent, teacher, child | Strengths and Difficulties Questionnaire (SDQ) | 3, 5, 7, 11, and 14 years | Sweeps 2-6 | At ages 7, 11, and 14, children self-reported on peer victimisation (e.g., “how often do other children hurt you or pick on you on purpose?”). The victimisation items from the SDQ were used for parent reports at ages 3, 5, 7, and 14, and teacher reports at ages 7 and 11 (e.g., “child picked on or bullied by other children”). Scores were dichotomised (most days/about once a week = 1 vs once a month/every few months/less often/never = 0). If children were reported by at least one informant to have experienced peer victimisation for at least 1 out of 5 sweeps, peer victimisation was coded as present. |
| Verbal victimisation | Child | Victimisation questionnaire | 14 years | Sweep 6 | Children self-reported whether they had ever been insulted, threatened, or shouted at by anyone in the past 12 months. Scores were dichotomised (yes = 1 vs no = 0). |
| Physical victimisation | Child | Victimisation questionnaire | 14 years | Sweep 6 | Children self-reported whether anyone had ever been physically violent (e.g., “pushed, shoved, hit, slapped or punched you”), or used a weapon against them in the past 12 months. Scores were dichotomised (yes = 1 vs no = 0). |
| Theft victimisation | Child | Victimisation questionnaire | 14 years | Sweep 6 | Children self-reported whether anyone had ever stolen something from them (e.g., mobile phone, money) in the past 12 months. Scores were dichotomised (yes = 1 vs no = 0). |
| Sexual victimisation | Child | Victimisation questionnaire | 14 years | Sweep 6 | Children self-reported whether anyone had ever sexually assaulted them in the past 12 months. Scores were dichotomised (yes = 1 vs no = 0). |
| Low cognitive stimulation | Parent | Parenting questionnaire | 3, 5, 7, and 11 years | Sweeps 2-5 | Cognitive stimulation was measured using several parent-reported items (e.g., “how often do you read to the child?”, “how often do you teach child counting?”) which were dichotomised (not at all/on special occasions = 1 vs once a week/several times a week/every day = 0). If cognitive stimulation was reported as low for at least 1 out of 4 sweeps, low cognitive stimulation was coded as present. |
| Neighbourhood deprivation | Parent | Neighbourhood questionnaire | 9 months | Sweep 1 | Neighbourhood deprivation was measured using several parent-reported items on the neighbourhood (e.g., “noisy neighbours”), cleanliness (e.g., “how common are rubbish/litter in area”), and access (e.g., “poor public transport”). Scores were dichotomised (very dissatisfied/fairly dissatisfied = 1 vs fairly satisfied/very satisfied = 0) and then summed. If more than half of the items were reported as unsatisfactory, neighbourhood deprivation was coded as present. |
| Unsafe home area | Parent | Housing questionnaire | 3, 5, and 11 years | Sweeps 2, 3, 5 | Home area safety was measured using several parent-reported items (e.g., “is this a good area to bring up a child?”, “how safe is it to walk/play in this area?”) which were dichotomised (very unsafe/fairly unsafe = 1 vs fairly safe/very safe = 0). If home area was reported as unsafe for at least 1 out of 3 sweeps, unsafe home area was coded as present. |
| Low household income | Parent | OECD Income Weighted Quintiles | 9 months, 3, 5, 7, 11, and 14 years | Sweeps 1-6 | Household equivalised income was measured using the OECD Income Weighted Quintiles. Scores were dichotomised (lowest quintile = 1 vs second/third/fourth/highest quintile = 0). If household income was reported as the lowest quintile for at least 1 out of 6 sweeps, low household income was coded as present. |
| Internalising symptoms | Child | Strengths and Difficulties Questionnaire (SDQ) | 17 years | Sweep 7 | Children self-reported on a three-point scale (0 = not true, 1 = somewhat true, 2 = certainly true) if they displayed: emotional problems (e.g., “I am often unhappy, down-hearted or tearful”) and peer problems (e.g., “other children pick on me or bully me”). Scores were summed across the internalising subscales and then standardised as a composite measure of internalising symptoms. |
| Externalising symptoms | Child | Strengths and Difficulties Questionnaire (SDQ) | 17 years | Sweep 7 | Children self-reported on a three-point scale (0 = not true, 1 = somewhat true, 2 = certainly true) if they displayed: conduct problems (e.g., “I get very angry and often lose my temper”) and hyperactivity/inattention (e.g., “I am restless, I cannot sit still for long”). Scores were summed across the externalising subscales and then standardised as a composite measure of externalising symptoms. |

**Table S2.** Criteria for deriving exposure and outcome measures from the Adolescent Brain Cognitive Development Study.

| Measure | Informant | Assessment type | Exposure period | Assessment  phase | Criteria for coding measure as present |
| --- | --- | --- | --- | --- | --- |
| Parental psychopathology | Parent | Family History Assessment, Adult Self-Report (ASR) | 0-9/10y,  11/12y | Baseline,  2y follow-up | Parents reported whether they had ever suffered from depression, manic episodes, psychotic experiences for more than 6 months, attempted or committed suicide in the Family History Assessment. If family history of psychopathology was present for either biological father or mother, or if parents scored above the clinical cut-off (ASR > 63; Achenbach & Rescorla, 2003) for depression, anxiety, or ADHD for at least 1 assessment, parental psychopathology was coded as present. |
| Parental alcohol abuse | Parent | Family History Assessment | 0-9/10y | Baseline | Parents reported whether they ever had any problems due to alcohol (e.g., marital problems, work problems, arrests/DUI, in a treatment programme, isolated self/caused arguments/drunk a lot). If family history of alcohol abuse was present for either biological father or mother, parental alcohol abuse was coded as present. |
| Parental drug abuse | Parent | Family History Assessment, Adult Self-Report (ASR) | 0-9/10y,  11/12y | Baseline,  2y follow-up | Parents reported whether they ever had any problems due to drugs (e.g., marital problems, work problems, arrests/DUI, in a treatment programme, isolated self/caused arguments/high a lot). If family history of drug abuse was present for either biological father or mother, or if parents reported using drugs multiple times weekly in the past 6 months in the ASR for at least 1 assessment, parental drug abuse was coded as present. |
| Parental separation | Parent | Parent Demographics Survey, Longitudinal Parent Demographics Survey, Parent Life Events | 0-9/10y,  10/11y,  11/12y | Baseline,  1y follow-up,  2y follow-up | Parents reported whether they had separated or divorced, or if their current partner was not the child’s biological or adoptive parent. If any instance of parental separation was reported for at least 1 assessment, parental separation was coded as present. |
| Domestic violence | Parent | KSADS Traumatic Events | 0-9/10y,  11/12y | Baseline,  2y follow-up | Parents reported whether their child had ever witnessed the grown-ups in the home push, shove or hit one another. If parents reported any instance of domestic violence for at least 1 assessment, domestic violence was coded as present. |
| Parental criminality | Parent | Parent Life Events | 0-10/11y,  11/12y | 1y follow-up,  2y follow-up | Parents reported whether they had ever got into trouble with the law or went to jail, or a family member had been arrested. If any instance of parental criminality was reported for at least 1 assessment, parental criminality was coded as present. |
| Peer victimisation | Child | Peer Experiences Questionnaire | 11/12y | 2y follow-up | Children reported whether they had been victimised by peers in the past year (e.g., kids left me out; chased me like trying to hurt me; spread rumours about me; did not invite me to party; left me out; gossiped about me; threatened to hurt/beat me; said mean things about me; hit/kicked/pushed me). Scores were dichotomised (a few times a week /once a week = 1 vs a few times/once or twice/never = 0). If children reported at least 1 type of victimisation, peer victimisation was coded as present. |
| Cyber victimisation | Child | Cyber Bully Questionnaire | 11/12y | 2y follow-up | Children reported whether they had experienced cybervictimisation, and if they had, how often they had been cyberbullied in the past year. Scores were dichotomised (≥ 10 times in the past 12 months = 1). If children reported at least 1 experience of cybervictimisation, cybervictimisation was coded as present. |
| Physical abuse | Parent | KSADS Traumatic Events | 0-9/10y,  11/12y | Baseline,  2y follow-up | Parents reported whether their child had ever been shot, stabbed, beaten brutally, or beaten to the point of having bruises by a grown-up in the home. If parents reported any instance of physical abuse for at least 1 assessment, physical abuse was coded as present. |
| Emotional abuse | Parent | KSADS Traumatic Events | 0-9/10y,  11/12y | Baseline,  2y follow-up | Parents reported whether a non-family member or family member had ever threatened to kill their child. If parents reported any instance of emotional abuse for at least 1 assessment, emotional abuse was coded as present. |
| Sexual abuse | Parent | KSADS Traumatic Events | 0-9/10y,  11/12y | Baseline,  2y follow-up | Parents reported whether a grown-up in the home or an adult outside the family had ever touched their child in their privates, had their child touch the adult’s privates, or if the adult did other sexual things to their child, or if a peer had ever forced their child to do something sexually. If parents reported any instance of sexual abuse for at least 1 assessment, sexual abuse was coded as present. |
| Emotional neglect | Child | Children’s Report of Parental Behavioural Inventory | 0-9/10y,  10/11y | Baseline,  1y follow-up | Children rated their caregiver’s behaviour (e.g., believes in showing love for me; makes me feel better when upset; or when talking over worries; is easy to talk to; smiles at me very often). Scores were dichotomised (not like him/her = 1 vs somewhat/a lot like him/her = 0) and then if children reported 2 or more items (cut-off score ≥ 2; Baldwin et al., 2023) for at least 1 assessment, emotional neglect was coded as present. |
| Accident requiring medical attention | Parent | KSADS Traumatic Events | 0-9/10y,  11/12y | Baseline,  2y follow-up | Parents reported whether their child had ever been in a car accident or another significant accident for which their child or another person needed medical attention. If parents reported any instance of an accident for at least 1 assessment, this was coded as present. |
| Natural disaster | Parent | KSADS Traumatic Events | 0-9/10y,  11/12y | Baseline,  2y follow-up | Parents reported whether their child had ever witnessed or been caught in a fire or natural disaster that caused significant property damage or personal injury. If parents reported any instance of a natural disaster for at least 1 assessment, this was coded as present. |
| Community violence | Parent | KSADS Traumatic Events | 0-9/10y,  11/12y | Baseline,  2y follow-up | Parents reported whether their child had ever witnessed an act of terrorism, death or mass destruction in a war zone, or someone shot or stabbed in the community. If parents reported any instance of community violence for at least 1 assessment, community violence was coded as present. |
| Bereavement | Parent | KSADS Traumatic Events | 0-9/10y,  11/12y | Baseline,  2y follow-up | Parents reported whether their child had ever learned about the sudden unexpected death of a loved one. If parents reported any instance of bereavement for at least 1 assessment, bereavement was coded as present. |
| Unsafe neighbourhood | Parent, Child | Parent and Youth Neighbourhood Safety/Crime Survey | 9/10y,  10/11y,  11/12y | Baseline,  1y follow-up,  2y follow-up | Parents and children reported neighbourhood safety (e.g., feel safe walking in my neighbourhood; violence is not a problem; my neighbourhood is safe from crime). Scores were dichotomised (strongly disagree = 1 vs disagree/neutral/agree/strongly agree = 0). If either parent or child reported the neighbourhood as unsafe for at least 1 assessment, unsafe neighbourhood was coded as present. |
| Low household income | Parent | Parent Demographics Survey | 9/10y,  10/11y,  11/12y | Baseline,  1y follow-up,  2y follow-up | Parents reported their total combined family income for the past 12 months. Scores were dichotomised according to guidelines from the US Census Bureau (lowest quintile households had ≤ $28,007 income = 1; Semega & Kollar, 2022). If household income was reported as the lowest quintile for at least 1 assessment, low household income was coded as present. |
| Internalising symptoms | Parent | Child Behaviour Checklist (CBCL) | 12/13y | 3y follow-up | Parents reported on a three-point scale (0 = absent, 1 = occurs sometimes, 2 = occurs often) if their child displayed: anxious/depressed behaviour (e.g., cries a lot), withdrawn/depressed behaviour (e.g., there is very little he/she enjoys), and somatic complaints (e.g., nightmares). Scores were summed across the internalising subscales and then standardised as a composite measure of internalising symptoms. |
| Externalising symptoms | Parent | Child Behaviour Checklist (CBCL) | 12/13y | 3y follow-up | Parents reported on a three-point scale (0 = absent, 1 = occurs sometimes, 2 = occurs often) if their child displayed: rule-breaking behaviour (e.g., drinks alcohol without parents’ approval), aggressive behaviour (e.g., argues a lot), and attention problems (e.g., fails to finish things he/she starts). Scores were summed across the externalising subscales and then standardised as a composite measure of externalising symptoms. |

**Figure S1.** Flowchart of participants in the Millennium Cohort Study.

Participants at MCS1 age 9 months survey

N = 18,552

Sample attrition N = 2,962

Participants at MCS2 age 3 survey

N = 15,590

Sample attrition N = 344

Participants at MCS3 age 5 survey

N = 15,246

Sample attrition N = 1,389

Participants at MCS4 age 7 survey

N = 13,857

Sample attrition N = 570

Participants at MCS5 age 11 survey

N = 13,287

Sample attrition N = 1,561

Participants at MCS6 age 14 survey

N = 11,726

Sample attrition N = 1,101

Participants at MCS7 age 17 survey

N = 10,625

Participants with missing data on ACEs and psychopathology

N = 4,032

Participants with complete data on ACEs and psychopathology

N = 6,502

# Table S3. Missing data per variable before multiple imputation in the MCS.

| *Variable* | *Complete n* | *Missing n* | *Missing* % |
| --- | --- | --- | --- |
| Poor parental mental health | 18,312 | 227 | 1.22 |
| Frequent parental alcohol use | 18,521 | 18 | 0.10 |
| Parental drug use | 15,574 | 2,965 | 15.99 |
| Single parent | 18,521 | 18 | 0.10 |
| Unhappy parental relationship | 16,383 | 2,156 | 11.63 |
| Domestic violence | 16,233 | 2,306 | 12.44 |
| Harsh parental discipline | 15,163 | 3,376 | 18.21 |
| Parental smacking | 15,118 | 3,421 | 18.45 |
| Negative home environment | 13,863 | 4,676 | 25.22 |
| Peer victimisation | 16,420 | 2,119 | 11.43 |
| Verbal victimisation | 10,787 | 7,752 | 41.81 |
| Physical victimisation | 10,786 | 7,753 | 41.82 |
| Theft victimisation | 10,782 | 7,757 | 41.84 |
| Sexual victimisation | 10,781 | 7,758 | 41.85 |
| Low household income | 18,513 | 26 | 0.14 |
| Neighbourhood deprivation | 17,844 | 695 | 3.75 |
| Unsafe home area | 16,351 | 2,188 | 11.80 |
| Low cognitive stimulation | 16,377 | 2,162 | 11.66 |
| Internalising symptoms | 9,398 | 9,141 | 49.31 |
| Externalising symptoms | 9,399 | 9,140 | 49.30 |

**Figure S2.** Flowchart of participants in the ABCD Study.

Participants at baseline age 9/10

N = 11,876

Sample attrition N = 651

Participants at year 1 follow-up age 10/11

N = 11,225

Sample attrition N = 811

Participants at year 2 follow-up age 11/12

N = 10,414

Sample attrition N = 4,163

Participants at year 3 follow-up age 12/13

N = 6,251

Participants with missing data on ACEs and psychopathology

N = 539

Participants with complete data on ACEs and psychopathology

N = 5,660

**Table S4.** Missing data per variable before multiple imputation in the ABCD Study.

| *Variable* | *Complete n* | *Missing n* | *Missing* % |
| --- | --- | --- | --- |
| Physical abuse | 11,836 | 40 | 0.34 |
| Emotional abuse | 11,836 | 40 | 0.34 |
| Sexual abuse | 11,836 | 40 | 0.34 |
| Domestic violence | 11,836 | 40 | 0.34 |
| Accident requiring medical attention | 11,836 | 40 | 0.34 |
| Natural disaster | 11,836 | 40 | 0.34 |
| Community violence | 11,836 | 40 | 0.34 |
| Bereavement | 11,836 | 40 | 0.34 |
| Emotional neglect | 11,876 | 0 | 0 |
| Parental psychopathology | 11,784 | 92 | 0.77 |
| Parental alcohol abuse | 11,876 | 0 | 0 |
| Parental drug abuse | 11,607 | 269 | 2.27 |
| Parental criminality | 11,446 | 430 | 3.62 |
| Parental separation | 11,409 | 467 | 3.93 |
| Peer victimisation | 10,392 | 1,484 | 12.50 |
| Cyber victimisation | 10,361 | 1,515 | 12.76 |
| Unsafe neighbourhood | 11,441 | 435 | 3.66 |
| Low household income | 10,607 | 1,269 | 10.69 |
| Internalising symptoms | 6,169 | 5,707 | 48.05 |
| Externalising symptoms | 6,169 | 5,707 | 48.05 |

**Appendix S2.** Exploratory factor analysis (EFA) on the MCS complete sample.

First, we examined the factorability of the ACE measures. Bartlett’s test of sphericity was significant, χ^2^(153) = 69,222.13, p<.001, indicating the presence of patterned relationships among the ACE measures. The Kaiser-Meyer-Olkin (KMO) measure of sampling adequacy was acceptable (KMO = 0.79), indicating the presence of latent factors.

We estimated the tetrachoric correlations among the 18 ACEs and plotted the tetrachoric correlation matrix with hierarchical clustering to group together similar measures. As there are visible clusters of between-measure correlations (e.g., between low household income and neighbourhood deprivation, and frequent parental alcohol use and parental drug use), it is evident there are at least two latent factors underlying the ACE measures (Figure S3).

**Figure S3.** Correlation matrix depicting tetrachoric correlations between ACEs in the MCS complete sample.

*Note.* Darker circles represent stronger correlations (blue for positive, red for negative).

Parallel analysis was used to determine how many factors should be retained in the exploratory factor analysis. We conducted parallel analysis with 1,000 Monte-Carlo simulations for both principal components (PC) and principal axis factoring (FA). As PC is a data reduction method that does not distinguish between shared and unique variance to reveal the underlying factor structure, we consulted the FA solution which recommended the optimal number of factors to retain was five (Figure S4).

Nevertheless, as FA parallel analysis is liable to selecting too many factors, Lim and Jahng (2019) recommended that parallel analysis should be regarded as a guide to factor extraction, not a fixed estimate. Thus, we looked to additional criteria. According to Cattell’s scree test (Cattell, 1966), the scree plot indicates three to four factors before the point of inflexion.

Altogether, parallel analysis and Cattell’s scree test recommended the extraction of three to five factors. The cumulative risk model assumes that ACEs would load onto a single latent factor, while DMAP proposes that ACEs load onto two factors of threat and deprivation. Thus, we conducted EFA to fit one, two, three, four, and five-factor models.

**Figure S4.** Parallel analysis scree plot generated after 1,000 simulations on the MCS complete sample.

*Note.* For both PC and FA, there were five factors generated above the 99^th^ percentile (represented as error bars connected by red dashed lines).

Model fit indices for one through five-factor models are presented in Table S5. As predicted by the parallel analysis, the five-factor model had the best fit according to the indices, followed by the four-factor, three-factor, two-factor, and one-factor models.

**Table S5.** EFA fit indices for one through five-factor models for the MCS complete sample.

| Factors | χ^2^ | RMSEA [90% CI] | RMSR | TLI | BIC | χ^2^ *p*-value |
| --- | --- | --- | --- | --- | --- | --- |
| 1 | 43735.73 | 0.132 [0.131, 0.133] | 0.12 | 0.31 | 42409.00 | <.001 |
| 2 | 19078.33 | 0.093 [0.092, 0.094] | 0.07 | 0.65 | 17918.67 | <.001 |
| 3 | 11399.50 | 0.077 [0.076, 0.078] | 0.05 | 0.76 | 10397.08 | <.001 |
| 4 | 7404.13 | 0.067 [0.066, 0.069] | 0.03 | 0.82 | 6549.12 | <.001 |
| 5 | 5431.04 | 0.063 [0.062, 0.064] | 0.03 | 0.84 | 4713.62 | <.001 |

*Note.* RMSEA = root mean square error of approximation; RMSR = root mean square residual (RMSR); TLI = Tucker-Lewis index (TLI); BIC = Bayesian Information Criterion.

Criteria for a good fit are as follows: RMSEA < 0.06; RMSR < 0.08; TLI > 0.95.

As the likelihood of the model increases, the BIC decreases; thus, a lower BIC indicates better fit.

We evaluated the best fitting model according to absolute and relative fit indices (RMSEA < 0.06, RMSR < 0.08, and TLI > 0.95 indicated good fit; Hu & Bentler, 1999). We also considered if the best fitting model had the “cleanest” factor structure: factor loadings equal to or more than 0.30, with no or few item cross loadings (Costello & Osborne, 2005).

The one-factor model (Figure S5) indicated poor fit indices compared to the rest of the models, and two ACE measures (low cognitive stimulation and sexual victimisation) had low loadings of 0.20. Parental smacking, frequent parental alcohol use, harsh parental discipline, and unhappy parental relationship did not load onto the one-factor model. As all ACE measures did not load onto a single factor, there was a lack of evidence for the cumulative risk model.

The two-factor model (Figure S6) had better fit indices than the one-factor model. Harsh parental discipline, parental smacking, and unhappy parental relationship did not load onto the two-factor model. Although there appeared to be two factors of threat/deprivation-related events and victimisation, there was no distinction between threat and deprivation ACEs to support DMAP.

The three-factor model (Figure S7) had better fit indices than the two-factor model, with all items loading onto three factors. The second and third factor were moderately correlated (*r =* 0.30) but their respective ACE measures loaded onto distinct dimensions.

The four-factor model (Figure S8) was the second-best fitting model in terms of fit indices, with all loadings equal to or above 0.30. The first three factors were moderately correlated with each other (*r =* 0.20), but their respective ACE measures loaded onto distinct dimensions.

The five-factor model (Figure S9) demonstrated the best fit indices, but the third factor consisted of only one item (parental drug use), indicating model instability. All five factors were correlated with each other (*r* = 0.20-0.30).

Overall consideration of the fit indices and factor structure suggested the four-factor model fit the MCS data optimally. The four factors were labelled deprivation, victimisation, parental threat, and parental discipline. After selecting the optimal model, we extracted factor scores and tested the associations between each factor and adolescent psychopathology.

**Figure S5.** One-factor model for the MCS complete sample.

**Figure S6.** Two-factor model for the MCS complete sample.

*Note.* Notably, frequent parental alcohol use correlated negatively with the first factor, which comprised of deprivation-related ACE measures. This might reflect the pattern of wealthier families affording higher alcohol consumption.

**Figure S7.** Three-factor model for the MCS complete sample.

**Figure S8.** Four-factor model for the MCS complete sample.

**Figure S9.** Five-factor model for the MCS complete sample.

*Note.* Notably, frequent parental alcohol use correlated negatively with the first factor, which comprised of deprivation-related ACE measures. This might reflect the pattern of wealthier families affording higher alcohol consumption.

**Appendix S3.** Exploratory factor analysis (EFA) on the MCS imputed sample.

EFA fit indices for the MCS imputed sample are presented in Table S6, which were broadly consistent with the EFA fit indices for the MCS complete sample (Table S5).

**Table S6.** EFA fit indices for one through five-factor models for the MCS imputed sample.

| Factors | χ^2^ | RMSEA [90% CI] | RMSR | TLI | BIC | χ^2^ *p*-value |
| --- | --- | --- | --- | --- | --- | --- |
| 1 | 60737.40 | 0.156 [0.155, 0.157] | 0.15 | 0.32 | 59410.67 | <.001 |
| 2 | 28746.84 | 0.114 [0.113, 0.116] | 0.08 | 0.63 | 27587.18 | <.001 |
| 3 | 13525.57 | 0.084 [0.083, 0.085] | 0.05 | 0.80 | 12523.15 | <.001 |
| 4 | 12031.58 | 0.086 [0.085, 0.087] | 0.03 | 0.80 | 11176.58 | <.001 |
| 5 | 7767.37 | 0.075 [0.074, 0.077] | 0.03 | 0.84 | 7049.95 | <.001 |

*Note.* RMSEA = root mean square error of approximation; RMSR = root mean square residual (RMSR); TLI = Tucker-Lewis index (TLI); BIC = Bayesian Information Criterion.

Criteria for a good fit are as follows: RMSEA < 0.06; RMSR < 0.08; TLI > 0.95.

As the likelihood of the model increases, the BIC decreases; thus, a lower BIC indicates better fit.

Plotting the factor loadings of the four-factor model for the MCS imputed sample (Figure S10) revealed identical factors of deprivation, victimisation, parental threat, and parental discipline as the complete sample (Figure S8). Thus, the imputed sample and complete sample produced consistent results despite their differences in missingness, demonstrating the validity of the four-factor model of deprivation, victimisation, parental threat, and parental discipline.

**Figure S10.** Four-factor model for the MCS imputed sample.

**Appendix S4.** Exploratory factor analysis (EFA) on the ABCD complete sample.

First, we examined the factorability of the ACE measures. Bartlett’s test of sphericity was significant, χ^2^(153) = 69,200.17, p<.001, indicating the presence of patterned relationships among the ACE measures. The Kaiser-Meyer-Olkin (KMO) measure of sampling adequacy was acceptable (KMO = 0.79), indicating the presence of latent factors.

We estimated the tetrachoric correlations among the 18 ACEs and plotted the tetrachoric correlation matrix with hierarchical clustering to group together similar measures. As there are visible clusters of between-measure correlations (e.g., between physical abuse and emotional abuse, and parental alcohol abuse and parental drug abuse), it is evident there are at least two latent factors underlying the ACE measures (Figure S11).

**Figure S11.** Correlation matrix depicting tetrachoric correlations between ACEs in the ABCD complete sample.

*Note.* Darker circles represent stronger correlations (blue for positive, red for negative).

We conducted parallel analysis on the ABCD sample with 1,000 Monte-Carlo simulations for both principal components (PC) and principal axis factoring (FA). Parallel analysis recommended the optimal number of factors to retain was four (Figure S12). According to Cattell’s scree test, the scree plot indicates two to three factors before the point of inflexion.

Altogether, parallel analysis and Cattell’s scree test recommended the extraction of two to four factors. The cumulative risk model assumes that ACEs would load onto a single latent factor, while DMAP proposes that ACEs load onto two factors of threat and deprivation. Thus, we conducted EFA to fit one, two, three, and four-factor models.

**Figure S12.** Parallel analysis scree plot generated after 1,000 simulations on the ABCD complete sample.

*Note.* For both PC and FA, there were four factors generated above the 99^th^ percentile (represented as error bars connected by red dashed lines).

Model fit indices for one through four-factor models are presented in Table S7. As predicted by the parallel analysis, the four-factor model had the best fit according to the indices, followed by the three-factor, two-factor, and one-factor models.

**Table S7.** EFA fit indices for one through four-factor models for the ABCD complete sample.

| Factors | χ^2^ | RMSEA [90% CI] | RMSR | TLI | BIC | χ^2^ *p*-value |
| --- | --- | --- | --- | --- | --- | --- |
| 1 | 28188.57 | 0.132 [0.131, 0.134] | 0.10 | 0.54 | 26921.96 | <.001 |
| 2 | 18247.88 | 0.114 [0.112, 0.115] | 0.07 | 0.66 | 17140.77 | <.001 |
| 3 | 15672.67 | 0.113 [0.112, 0.115] | 0.06 | 0.66 | 14715.67 | <.001 |
| 4 | 8565.06 | 0.091 [0.089, 0.092] | 0.03 | 0.78 | 7748.80 | <.001 |

*Note.* RMSEA = root mean square error of approximation; RMSR = root mean square residual (RMSR); TLI = Tucker-Lewis index (TLI); BIC = Bayesian Information Criterion.

Criteria for a good fit are as follows: RMSEA < 0.06; RMSR < 0.08; TLI > 0.95.

As the likelihood of the model increases, the BIC decreases; thus, a lower BIC indicates better fit.

As with the MCS sample, we evaluated the best fitting model according to absolute and relative fit indices (RMSEA < 0.06, RMSR < 0.08, and TLI > 0.95 indicated good fit; Hu & Bentler, 1999). We also considered if the best fitting model had the “cleanest” factor structure: factor loadings equal to or more than 0.30, with no or few item cross loadings (Costello & Osborne, 2005).

The one-factor model (Figure S13) indicated poor fit indices compared to the rest of the models, and one ACE measure (peer victimisation) had a low loading of 0.20. Emotional neglect did not load onto the one-factor model. Given that all ACE measures did not load onto a single factor, there was a lack of evidence to support the cumulative risk model.

The two-factor model (Figure S14) had better fit indices than the one-factor model. Peer victimisation did not load onto the two-factor model. The first and second factors were correlated (*r =* 0.50) but their respective ACE measures loaded onto distinct dimensions. Although there appeared to be two factors of threat/deprivation-related events and traumatic events, there was no distinction between threat and deprivation ACEs to support DMAP.

The three-factor model (Figure S15) had better fit indices than the two-factor model, with all items loading onto three factors. The first and second factors were identical to the two-factor model, except that emotional neglect, peer victimisation and cyber victimisation loaded onto the third factor.

The four-factor model (Figure S16) demonstrated the best fit indices, with all loadings equal to or above 0.30. The first three factors were moderately correlated with each other (*r =* 0.30-0.40) but their respective ACE measures loaded onto distinct dimensions.

Overall consideration of the fit indices and factor structure criteria suggested the four-factor model fit the ABCD data optimally. The four factors were labelled traumatic events, parental threat, deprivation, and victimisation. After selecting the optimal model, we extracted factor scores and tested the associations between each factor and adolescent psychopathology.

**Figure S13.** One-factor model for the ABCD complete sample.

**Figure S14.** Two-factor model for the ABCD complete sample.

**Figure S15.** Three-factor model for the ABCD complete sample.

**Figure S16.** Four-factor model for the ABCD complete sample.

**Appendix S5.** Exploratory factor analysis (EFA) on the ABCD imputed sample.

EFA fit indices for the ABCD imputed sample are presented in Table S8, which were broadly consistent with the EFA fit indices for the ABCD complete sample (Table S7).

**Table S8.** EFA fit indices for one through four-factor models for the ABCD imputed sample.

| Factors | χ^2^ | RMSEA [90% CI] | RMSR | TLI | BIC | χ^2^ *p*-value |
| --- | --- | --- | --- | --- | --- | --- |
| 1 | 28986.87 | 0.134 [0.133, 0.135] | 0.10 | 0.53 | 27720.26 | <.001 |
| 2 | 18760.36 | 0.115 [0.114, 0.117] | 0.07 | 0.65 | 17653.25 | <.001 |
| 3 | 16056.97 | 0.115 [0.113, 0.116] | 0.06 | 0.65 | 15099.97 | <.001 |
| 4 | 8566.52 | 0.091 [0.089, 0.092] | 0.04 | 0.79 | 7750.27 | <.001 |

*Note.* RMSEA = root mean square error of approximation; RMSR = root mean square residual (RMSR); TLI = Tucker-Lewis index (TLI); BIC = Bayesian Information Criterion.

Criteria for a good fit are as follows: RMSEA < 0.06; RMSR < 0.08; TLI > 0.95.

As the likelihood of the model increases, the BIC decreases; thus, a lower BIC indicates better fit.

Plotting the factor loadings of the four-factor model for the ABCD imputed sample (Figure S17) revealed identical factors of deprivation, victimisation, parental threat, and traumatic events as the complete sample (Figure S16). Thus, the imputed sample and complete sample produced consistent results despite their differences in missingness, demonstrating the validity of the four-factor model of deprivation, victimisation, parental threat, and traumatic events.

**Figure S17.** Four-factor model for the ABCD imputed sample.

**Appendix S6.** Associations between adversity dimensions and adolescent psychopathology in the MCS complete sample.

Next, we examined the relationships between deprivation, victimisation, parental threat, and parental discipline with internalising and externalising symptoms at age 17. Before proceeding with the regression analyses, we examined the correlations between the four factors. Certain factors were moderately correlated with each other, for example: deprivation and parental threat (*r =* 0.21), and victimisation and parental threat (*r* = 0.22). The remaining factors showed little to no correlation with each other: parental threat and parental discipline (*r* = 0.13), victimisation and parental discipline (*r* = 0.11), deprivation and victimisation (*r* = 0.07), and deprivation and parental discipline (*r* = 0.01).

Results from the unadjusted univariate regression models are reported below in text, while results from the adjusted models are presented in Tables S9-S10.

Univariate analyses revealed that all four factors were associated with adolescent psychopathology in different ways. There was a significant association between deprivation and internalising symptoms (*β* = 0.12, 95% CI = 0.10 – 0.15, *p* <.001), as well as externalising symptoms (*β* = 0.10, 95% CI = 0.08 – 0.13, *p* <.001). After adjusting for sex and race, these associations remained significant.

There were significant associations between victimisation and internalising symptoms (*β* = 0.23, 95% CI = 0.21 – 0.25, *p* <.001) and externalising symptoms (*β* = 0.22, 95% CI = 0.20 – 0.24, *p* <.001), both of which remained significant after adjusting for covariates.

There was a small association between parental threat and internalising symptoms (*β* = 0.10, 95% CI = 0.08 – 0.13, *p* <.001), as well as externalising symptoms (*β* = 0.08, 95% CI = 0.05 – 0.10, *p* <.001). These remained significantly associated after adjusting for covariates.

There was initially no association between parental discipline and internalising symptoms (*β* = 0.0001, 95% CI = -0.02 – 0.02, *p* =.992), but parental discipline was associated with externalising symptoms (*β* = 0.12, 95% CI = 0.09 – 0.14, *p* <.001). After covariate adjustment, parental discipline was associated with both internalising and externalising symptoms.

In the multivariate adjusted model, parental threat was no longer associated with internalising symptoms (*β* = 0.01, 95% CI = -0.01 – 0.04, *p* = .244) or externalising symptoms (*β* = 0.003, 95% CI = -0.02 – 0.03, *p* = .812). Additionally, parental discipline (*β* = 0.01, 95% CI = -0.01 – 0.03, *p* = .461) was no longer associated with internalising symptoms.

Compared to the other three factors, victimisation appeared to be the most strongly associated with internalising symptoms (*β* = 0.23, 95% CI = 0.21 – 0.26, *p* <.001) and externalising symptoms (*β* = 0.19, 95% CI = 0.17 – 0.22, *p* <.001). Next, deprivation demonstrated increased risk for internalising symptoms (*β* = 0.12, 95% CI = 0.10 – 0.15, *p* <.001) and externalising symptoms (*β* = 0.10, 95% CI = 0.07 – 0.12, *p* <.001). After accounting for the other three factors, parental discipline only remained associated with increased risk for externalising symptoms (*β* = 0.09, 95% CI = 0.06 – 0.11, *p* <.001).

**Table S9.** Adjusted associations between ACE dimensions and internalising symptoms in the MCS complete sample.

|  | | Internalising symptoms at age 17 | | | | |
| --- | --- | --- | --- | --- | --- | --- |
|  | | Model 1:  Deprivation ~ Internalising | Model 2:  Victimisation ~ Internalising | Model 3:  Parental Threat ~ Internalising | Model 4:  Parental Discipline ~ Internalising | Model 5:  Deprivation + Victimisation + Parental Threat + Parental Discipline ~ Internalising |
|  | | *β* (95% CI) | *β* (95% CI) | *β* (95% CI) | *β* (95% CI) | *β* (95% CI) |
|  |  | | | | | |
| *ACEs* | |  |  |  |  |  |
| Deprivation | | 0.15^***^ (0.12, 0.17) |  |  |  | 0.12^***^ (0.10, 0.15) |
| Victimisation | |  | 0.25^***^ (0.23, 0.27) |  |  | 0.23^***^ (0.21, 0.26) |
| Parental Threat | |  |  | 0.09^***^ (0.07, 0.12) |  | 0.01 (-0.01, 0.04) |
| Parental Discipline | |  |  |  | 0.04^***^ (0.01, 0.06) | 0.01 (-0.01, 0.03) |
| *Covariates* | |  |  |  |  |  |
| Sex (ref: male) | |  |  |  |  |  |
| Female | | 0.51^***^ (0.46, 0.55) | 0.55^***^ (0.50, 0.59) | 0.51^***^ (0.46, 0.55) | 0.52^***^ (0.47, 0.57) | 0.54^***^ (0.50, 0.59) |
| Ethnicity (ref: White) | |  |  |  |  |  |
| Black or Black British | | -0.41^***^ (-0.57, -0.24) | -0.32^***^ (-0.48, -0.16) | -0.30^***^ (-0.47, -0.14) | -0.32^***^ (-0.48, -0.15) | -0.40^***^ (-0.56, -0.24) |
| Indian | | -0.29^***^ (-0.44, -0.14) | -0.20^***^ (-0.35, -0.05) | -0.20^***^ (-0.36, -0.05) | -0.25^***^ (-0.40, -0.09) | -0.24^***^ (-0.38, -0.09) |
| Mixed | | 0.06 (-0.09, 0.22) | 0.07 (-0.09, 0.22) | 0.07 (-0.09, 0.23) | 0.10 (-0.06, 0.26) | 0.03 (-0.12, 0.19) |
| Other (inc. Chinese) | | -0.39^***^ (-0.62, -0.16) | -0.23^**^ (-0.46, -0.01) | -0.31^***^ (-0.54, -0.08) | -0.32^***^ (-0.56, -0.09) | -0.30^***^ (-0.53, -0.08) |
| Pakistani and Bangladeshi | | -0.39^***^ (-0.51, -0.28) | -0.16^***^ (-0.26, -0.05) | -0.21^***^ (-0.32, -0.10) | -0.25^***^ (-0.36, -0.14) | -0.28^***^ (-0.39, -0.17) |

*Note.* Predictors (deprivation, victimisation, parental threat, and parental discipline) and outcomes (internalising and externalising symptoms) were standardised.

*β* = standardised regression coefficient, CI = confidence interval.

**p* <.05; ***p* <.01; ****p* <.001

**Table S10.** Adjusted associations between ACE dimensions and externalising symptoms in the MCS complete sample.

|  | | Externalising symptoms at age 17 | | | | |
| --- | --- | --- | --- | --- | --- | --- |
|  | | Model 1:  Deprivation ~ Externalising | Model 2:  Victimisation ~ Externalising | Model 3:  Parental Threat ~ Externalising | Model 4:  Parental Discipline ~ Externalising | Model 5:  Deprivation + Victimisation + Parental Threat + Parental Discipline ~ Externalising |
|  | | *β* (95% CI) | *β* (95% CI) | *β* (95% CI) | *β* (95% CI) | *β* (95% CI) |
|  |  | | | | | |
| *ACEs* | |  |  |  |  |  |
| Deprivation | | 0.12^***^ (0.09, 0.14) |  |  |  | 0.10^***^ (0.07, 0.12) |
| Victimisation | |  | 0.21^***^ (0.19, 0.24) |  |  | 0.19^***^ (0.17, 0.22) |
| Parental Threat | |  |  | 0.08^***^ (0.05, 0.10) |  | 0.003 (-0.02, 0.03) |
| Parental Discipline | |  |  |  | 0.11^***^ (0.09, 0.14) | 0.09^***^ (0.06, 0.11) |
| *Covariates* | |  |  |  |  |  |
| Sex (ref: male) | |  |  |  |  |  |
| Female | | -0.16^***^ (-0.21, -0.11) | -0.13^***^ (-0.18, -0.08) | -0.16^***^ (-0.21, -0.11) | -0.13^***^ (-0.18, -0.08) | -0.11^***^ (-0.16, -0.06) |
| Ethnicity (ref: White) | |  |  |  |  |  |
| Black or Black British | | -0.20^**^ (-0.37, -0.03) | -0.13 (-0.29, 0.04) | -0.12 (-0.29, 0.05) | -0.14 (-0.31, 0.03) | -0.20^**^ (-0.37, -0.03) |
| Indian | | -0.06 (-0.22, 0.10) | 0.01 (-0.14, 0.17) | 0.01 (-0.15, 0.16) | -0.04 (-0.20, 0.11) | -0.04 (-0.19, 0.12) |
| Mixed | | -0.002 (-0.17, 0.16) | -0.001 (-0.16, 0.16) | 0.003 (-0.16, 0.17) | 0.03 (-0.13, 0.19) | -0.02 (-0.18, 0.14) |
| Other (inc. Chinese) | | -0.19 (-0.43, 0.05) | -0.06 (-0.29, 0.17) | -0.13 (-0.37, 0.11) | -0.16 (-0.39, 0.08) | -0.14 (-0.37, 0.09) |
| Pakistani and Bangladeshi | | -0.25^***^ (-0.36, -0.13) | -0.05 (-0.16, 0.06) | -0.10 (-0.21, 0.02) | -0.14^**^ (-0.25, -0.03) | -0.17^***^ (-0.28, -0.05) |

*Note.* Predictors (deprivation, victimisation, parental threat, and parental discipline) and outcomes (internalising and externalising symptoms) were standardised.

*β* = standardised regression coefficient, CI = confidence interval.

**p* <.05; ***p* <.01; ****p* <.001

We also tested for multicollinearity by calculating the variance inflation factor (VIF) and tolerance values for the multivariate model. VIF values were all around 1 and tolerance values were much greater than 0.10 (Tables S11-S12), indicating that multicollinearity was very unlikely.

**Table S11.** Variance inflation factor (VIF) for the multivariate model in the MCS complete sample.

| ACE Dimension | VIF [95% CI] | Tolerance [95% CI] |
| --- | --- | --- |
| Deprivation | 1.13 [1.10, 1.16] | 0.89 [0.86, 0.91] |
| Victimisation | 1.07 [1.05, 1.11] | 0.93 [0.90, 0.95] |
| Parental threat | 1.13 [1.10, 1.16] | 0.89 [0.86, 0.91] |
| Parental discipline | 1.05 [1.03, 1.08] | 0.96 [0.92, 0.97] |

**Table S12.** Variance inflation factor (VIF) for the multivariate model in the MCS imputed sample.

| ACE Dimension | VIF [95% CI] | Tolerance [95% CI] |
| --- | --- | --- |
| Deprivation | 1.16 [1.14, 1.18] | 0.86 [0.85, 0.87] |
| Victimisation | 1.11 [1.09, 1.13] | 0.90 [0.89, 0.91] |
| Parental threat | 1.24 [1.22, 1.26] | 0.81 [0.80, 0.82] |
| Parental discipline | 1.13 [1.11, 1.15] | 0.88 [0.87, 0.90] |

**Appendix S7.** Gender interactions in the MCS complete sample.

Notably, being female was associated with significantly increased risk for internalising symptoms (*β* = 0.54, 95% CI = 0.50 – 0.59, *p* = <.001) but decreased risk for externalising symptoms (*β* = -0.11, 95% CI = -0.16 – 0.06, *p* = <.001). Thus, we tested for interactions between sex and deprivation, victimisation, parental threat, and parental discipline. There were no significant interactions between sex and any of the four factors for externalising symptoms.

However, there was a small significant interaction between female sex and victimisation for internalising symptoms. Specifically, girls who experienced victimisation ACEs were at slightly higher risk for internalising symptoms than boys who experienced victimisation (*β* = 0.07, 95% CI = 0.02 – 0.11, *p* = <.01).

We then stratified the sample by gender and found that for boys, only deprivation (*β* = 0.11, 95% CI = 0.08 – 0.15, *p* <.001) and victimisation (*β* = 0.20, 95% CI = 0.17 – 0.23, *p* <.001) remained associated with internalising symptoms in the multivariate model. Boys who had experienced parental threat (*β* = -0.001, 95% CI = -0.03 – 0.03, *p* = .951) and parental discipline (*β* = -0.008, 95% CI = -0.04 – 0.02, *p* = .624) were not at increased risk for internalising symptoms.

For girls, only deprivation (*β* = 0.13, 95% CI = 0.10 – 0.17, *p* <.001) and victimisation (*β* = 0.27, 95% CI = 0.23 – 0.30, *p* <.001) remained associated with internalising symptoms in the multivariate model. Girls who had experienced parental threat (*β* = 0.03, 95% CI = -0.01 – 0.06, *p* = .154) and parental discipline (*β* = 0.03, 95% CI = -0.01 – 0.06, *p* = .102) were not at increased risk for internalising symptoms.

Overall, the gender stratified regression results remained consistent for boys and girls. Victimisation and deprivation significantly increased the risk for internalising symptoms, and victimisation demonstrated larger effect sizes than deprivation. As the gender interaction analyses were originally intended to be exploratory, we presented results for the total sample.

**Appendix S8.** Associations between adversity dimensions and adolescent psychopathology in the ABCD complete sample.

Next, we examined the relationships between traumatic events, parental threat, deprivation, and victimisation with internalising and externalising symptoms at age 12-13. Before proceeding with the regression analyses, we examined the correlations between the four factors. Certain factors were moderately correlated with each other, for example: traumatic events and parental threat (*r* = 0.41), traumatic events and deprivation (*r* = 0.28), and parental threat and deprivation (*r* = 0.27). The remaining factors showed little to no correlation with each other: traumatic events and victimisation (*r* = 0.14), parental threat and victimisation (*r* = 0.16), and deprivation and victimisation (*r* = 0.08).

Results from the unadjusted univariate regression models are reported below in text, while results from the adjusted models are presented in Tables S13-S14.

Univariate analyses revealed that all four factors were associated with adolescent psychopathology. There was a significant association between traumatic events and internalising symptoms (*β* = 0.14, 95% CI = 0.11 – 0.16, *p* <.001), as well as externalising symptoms (*β* = 0.16, 95% CI = 0.13 – 0.18, *p* <.001). After adjusting for sex and race, these associations remained significant.

There were slightly stronger associations between parental threat and internalising symptoms (*β* = 0.20, 95% CI = 0.17 – 0.22, *p* <.001) and externalising symptoms (*β* = 0.22, 95% CI = 0.20 – 0.25, *p* <.001), both of which remained significant after adjusting for covariates.

There was a small significant association between deprivation and internalising symptoms (*β* = 0.04, 95% CI = 0.01 – 0.07, *p* <.001), with a stronger association for deprivation and externalising symptoms (*β* = 0.12, 95% CI = 0.09 – 0.15, *p* <.001). These remained significantly associated after adjusting for covariates.

Victimisation was also associated with increased risk for internalising symptoms (*β* = 0.17, 95% CI = 0.14 – 0.19, *p* <.001) and externalising symptoms (*β* = 0.20, 95% CI = 0.17 – 0.22, *p* <.001), and continued being associated after covariate adjustment.

In the multivariate adjusted model, all four factors remained independently associated with increased risk for externalising symptoms. However, for internalising symptoms, the deprivation factor was no longer significantly associated (*β* = -0.02, 95% CI = -0.05 – 0.02, *p* = .337) after controlling for traumatic events, parental threat, and victimisation. Additionally, the association between deprivation and externalising symptoms barely met the minimum significance threshold (*β* = 0.03, 95% CI = 0.001 – 0.06, *p* = .042).

Compared to the other three factors, parental threat appeared to be the most strongly associated with internalising symptoms (*β* = 0.16, 95% CI = 0.13 – 0.18, *p* <.001) and externalising symptoms (*β* = 0.16, 95% CI = 0.14 – 0.19, *p* <.001). Next, the victimisation factor demonstrated similar effect sizes with increased risk for internalising symptoms (*β* = 0.13, 95% CI = 0.11 – 0.16, *p* <.001) and externalising symptoms (*β* = 0.16, 95% CI = 0.14 – 0.19, *p* <.001). After accounting for the other three factors, traumatic events also remained associated with internalising symptoms (*β* = 0.07, 95% CI = 0.04 – 0.09, *p* <.001) and externalising symptoms (*β* = 0.06, 95% CI = 0.03 – 0.09, *p* <.001).

**Table S13.** Adjusted associations between ACE dimensions and internalising symptoms in the ABCD complete sample.

|  | | Internalising symptoms at age 12-13 | | | | |
| --- | --- | --- | --- | --- | --- | --- |
|  | | Model 1:  Traumatic events ~ Internalising | Model 2:  Parental threat ~ Internalising | Model 3:  Deprivation ~ Internalising | Model 4:  Victimisation ~ Internalising | Model 5:  Traumatic events + Parental threat + Deprivation + Victimisation ~ Internalising |
|  | | *β* (95% CI) | *β* (95% CI) | *β* (95% CI) | *β* (95% CI) | *β* (95% CI) |
|  |  | | | | | |
| *ACEs* | |  |  |  |  |  |
| Traumatic events | | 0.14^***^ (0.11, 0.16) |  |  |  | 0.07^***^ (0.04, 0.09) |
| Parental threat | |  | 0.20^***^ (0.17, 0.22) |  |  | 0.16^***^ (0.13, 0.18) |
| Deprivation | |  |  | 0.06^***^ (0.03, 0.09) |  | -0.02 (-0.05, 0.02) |
| Victimisation | |  |  |  | 0.16^***^ (0.14, 0.19) | 0.13^***^ (0.11, 0.16) |
| *Covariates* | |  |  |  |  |  |
| Sex (ref: male) | |  |  |  |  |  |
| Female | | 0.17^***^ (0.11, 0.22) | 0.16^***^ (0.11, 0.22) | 0.17^***^ (0.12, 0.22) | 0.17^***^ (0.11, 0.22) | 0.16^***^ (0.11, 0.21) |
| Ethnicity (ref: White) | |  |  |  |  |  |
| American Indian/Alaska Native | | 0.14 (-0.01, 0.29) | 0.05 (-0.10, 0.20) | 0.17^**^ (0.02, 0.32) | 0.17^**^ (0.02, 0.32) | 0.05 (-0.10, 0.20) |
| Asian | | -0.14^**^ (-0.25, -0.03) | -0.12^**^ (-0.22, -0.01) | -0.15^***^ (-0.26, -0.04) | -0.13^**^ (-0.24, -0.02) | -0.10 (-0.21, 0.01) |
| Black/African American | | -0.20^***^ (-0.27, -0.12) | -0.22^***^ (-0.29, -0.14) | -0.21^***^ (-0.29, -0.13) | -0.15^***^ (-0.23, -0.07) | -0.20^***^ (-0.28, -0.12) |
| Native Hawaiian/Pacific Islander | | 0.07 (-0.33, 0.48) | -0.01 (-0.41, 0.39) | 0.07 (-0.34, 0.48) | 0.03 (-0.37, 0.44) | -0.04 (-0.43, 0.36) |
| Other Race | | 0.06 (-0.05, 0.18) | 0.04 (-0.07, 0.15) | 0.04 (-0.07, 0.15) | 0.08 (-0.03, 0.19) | 0.05 (-0.06, 0.16) |

*Note.* Predictors (traumatic events, parental threat, deprivation, and victimisation) and outcomes (internalising and externalising symptoms) were standardised.

*β* = standardised regression coefficient, CI = confidence interval.

**p* <.05; ***p* <.01; ****p* <.001

**Table S14.** Adjusted associations between ACE dimensions and externalising symptoms in the ABCD complete sample.

|  | | Externalising symptoms at age 12-13 | | | | |
| --- | --- | --- | --- | --- | --- | --- |
|  | | Model 1:  Traumatic events ~ Externalising | Model 2:  Parental threat ~ Externalising | Model 3:  Deprivation ~ Externalising | Model 4:  Victimisation ~ Externalising | Model 5:  Traumatic events + Parental threat + Deprivation + Victimisation ~ Externalising |
|  | | *β* (95% CI) | *β* (95% CI) | *β* (95% CI) | *β* (95% CI) | *β* (95% CI) |
|  |  | | | | | |
| *ACEs* | |  |  |  |  |  |
| Traumatic events | | 0.15^***^ (0.13, 0.18) |  |  |  | 0.06^***^ (0.03, 0.09) |
| Parental threat | |  | \| 0.22^***^ (0.19, 0.25) \|  \| \| --- \| --- \| |  |  | 0.16^***^ (0.14, 0.19) |
| Deprivation | |  |  | 0.11^***^ (0.08, 0.14) |  | 0.03^*^ (0.001, 0.06) |
| Victimisation | |  |  |  | 0.19^***^ (0.17, 0.22) | 0.16^***^ (0.14, 0.19) |
| *Covariates* | |  |  |  |  |  |
| Sex (ref: male) | |  |  |  |  |  |
| Female | | -0.27^***^ (-0.32, -0.22) | -0.27^***^ (-0.33, -0.22) | -0.26^***^ (-0.31, -0.21) | -0.27^***^ (-0.32, -0.22) | -0.28^***^ (-0.32, -0.23) |
| Ethnicity (ref: White) | |  |  |  |  |  |
| American Indian/Alaska Native | | 0.18^**^ (0.03, 0.33) | 0.09 (-0.06, 0.23) | 0.20^**^ (0.05, 0.35) | 0.21^***^ (0.06, 0.36) | 0.07 (-0.08, 0.22) |
| Asian | | -0.14^***^ (-0.25, -0.04) | -0.12^**^ (-0.22, -0.01) | -0.15^***^ (-0.26, -0.04) | -0.13^**^ (-0.24, -0.02) | -0.10 (-0.20, 0.01) |
| Black/African American | | 0.09^**^ (0.01, 0.16) | 0.06 (-0.01, 0.14) | 0.03 (-0.05, 0.11) | 0.14^***^ (0.06, 0.21) | 0.04 (-0.03, 0.12) |
| Native Hawaiian/Pacific Islander | | 0.16 (-0.24, 0.56) | 0.07 (-0.33, 0.47) | 0.15 (-0.25, 0.55) | 0.11 (-0.29, 0.51) | 0.03 (-0.36, 0.42) |
| Other Race | | 0.04 (-0.07, 0.15) | 0.02 (-0.09, 0.13) | -0.01 (-0.13, 0.10) | 0.06 (-0.05, 0.17) | -0.002 (-0.11, 0.11) |

*Note.* Predictors (traumatic events, parental threat, deprivation, and victimisation) and outcomes (internalising and externalising symptoms) were standardised.

*β* = standardised regression coefficient, CI = confidence interval.

**p* <.05; ***p* <.01; ****p* <.001

We also tested for multicollinearity by calculating the variance inflation factor (VIF) and tolerance values for the multivariate model. VIF values were all around 1 and tolerance values were much greater than 0.10 (Tables S15-S16), indicating that multicollinearity was very unlikely.

**Table S15.** Variance inflation factor (VIF) and tolerance values for the multivariate model for the ABCD complete sample.

| ACE Dimension | VIF [95% CI] | Tolerance [95% CI] |
| --- | --- | --- |
| Traumatic events | 1.25 [1.21, 1.29] | 0.80 [0.78, 0.83] |
| Parental threat | 1.27 [1.24, 1.32] | 0.79 [0.76, 0.81] |
| Deprivation | 1.27 [1.23, 1.31] | 0.79 [0.76, 0.81] |
| Victimisation | 1.03 [1.01, 1.08] | 0.97 [0.93, 0.99] |

**Table S16.** Variance inflation factor (VIF) and tolerance values for the multivariate model for the ABCD imputed sample.

| ACE Dimension | VIF [95% CI] | Tolerance [95% CI] |
| --- | --- | --- |
| Traumatic events | 1.26 [1.24, 1.29] | 0.79 [0.77, 0.81] |
| Parental threat | 1.25 [1.22, 1.27] | 0.80 [0.78, 0.82] |
| Deprivation | 1.31 [1.29, 1.34] | 0.76 [0.74, 0.78] |
| Victimisation | 1.04 [1.03, 1.07] | 0.96 [0.94, 0.97] |

**Appendix S9.** Gender interactions in the ABCD complete sample.

Notably, being female was associated with significantly increased risk for internalising symptoms (*β* = 0.16, 95% CI = 0.11 – 0.21, *p* <.001), but decreased risk for externalising symptoms (*β* = -0.28, 95% CI = -0.32 – -0.23, *p* <.001). Thus, we tested for interactions between sex and traumatic events, parental threat, deprivation, and victimisation. There were no significant interactions between sex and any of the four factors for internalising symptoms.

However, there was a small significant interaction between male sex and parental threat for externalising symptoms. Specifically, boys who experienced parental threat ACEs were at slightly higher risk for externalising symptoms than girls who experienced parental threat (*β* = 0.08, 95% CI = 0.02 – 0.14, *p* <.01).

We then stratified the sample by gender and found that for boys, only traumatic events (*β* = 0.07, 95% CI = 0.03 – 0.11, *p* <.01), parental threat (*β* = 0.20, 95% CI = 0.16 – 0.25, *p* <.001), and victimisation (*β* = 0.17, 95% CI = 0.13 – 0.20, *p* <.001) remained associated with externalising symptoms in the multivariate model. Boys who had experienced deprivation were not at increased risk for externalising symptoms (*β* = 0.01, 95% CI = -0.03 – 0.06, *p* = .590).

For girls, all four factors of traumatic events (*β* = 0.05, 95% CI = 0.02 – 0.09, *p* <.01), parental threat (*β* = 0.12, 95% CI = 0.09 – 0.16, *p* <.001), deprivation (*β* = 0.05, 95% CI = 0.01 – 0.09, *p* <.01), and victimisation (*β* = 0.16, 95% CI = 0.13 – 0.19, *p* <.001) were significantly associated with externalising symptoms in the multivariate model.

Overall, the gender stratified regression results remained consistent for boys and girls. Parental threat significantly increased the risk for externalising symptoms, and parental threat and victimisation demonstrated larger effect sizes than traumatic events and deprivation. As the gender interaction analyses were originally intended to be exploratory, we presented results for the total sample.

References

Achenbach, T.M. & Rescorla, L.A. (2003). Manual for the ASEBA Adult Forms and Profiles. University of Vermont, Research Center for Children, Youth and Families, Burlington, VT.

Adjei, N. K., Schlüter, D. K., Straatmann, V. S., Melis, G., Fleming, K. M., McGovern, R., Howard, L. M., Kaner, E., Wolfe, I., & Taylor-Robinson, D. C. (2022). Impact of poverty and family adversity on adolescent health: a multi-trajectory analysis using the UK Millennium Cohort Study. *The Lancet Regional Health - Europe*, *13*(100279), 100279. https://doi.org/10.1016/j.lanepe.2021.100279

Baldwin, J. R., Sallis, H. M., Schoeler, T., et al. (2023). A genetically informed Registered Report on adverse childhood experiences and mental health. *Nature Human Behaviour*, *7*(2), 269-290. doi:10.1038/s41562-022-01482-9

Bevilacqua, L., Kelly, Y., Heilmann, A., Priest, N., & Lacey, R. E. (2021). Adverse childhood experiences and trajectories of internalizing, externalizing, and prosocial behaviors from childhood to adolescence. *Child Abuse & Neglect*, *112*(104890), 1–13. https://doi.org/10.1016/j.chiabu.2020.104890

Cattell, R. B. (1966). The scree test for the number of factors. *Multivariate Behavioural Research, 1*, 245- 276.

Costello, A. B., & Osborne, J. W. (2005). Best practices in exploratory factor analysis: Four recommendations for getting the most from your analysis. *Practical Assessment, Research, and Evaluation*, *10*(7), 1–10. https://doi.org/10.7275/jyj1-4868

Hu, L., & Bentler, P. M. (1999). Cutoff criteria for fit indexes in covariance structure analysis: Conventional criteria versus new alternatives. *Structural Equation Modeling: A Multidisciplinary Journal*, *6*(1), 1–55. https://doi.org/10.1080/10705519909540118

Jessica Semega and Melissa Kollar, U.S. Census Bureau, Current Population Reports, P60-276, Income in the United States: 2021, U.S. Government Publishing Office, Washington, DC, September 2022.

Lim, S., & Jahng, S. (2019). Determining the number of factors using parallel analysis and its recent variants. *Psychological Methods*, *24*(4), 452–467. https://doi.org/10.1037/met0000230

Straatmann, V. S., Lai, E., Law, C., Whitehead, M., Strandberg-Larsen, K., & Taylor-Robinson, D. (2020). How do early-life adverse childhood experiences mediate the relationship between childhood socioeconomic conditions and adolescent health outcomes in the UK? *Journal of Epidemiology and Community Health*, *74*(11), 969–975. https://doi.org/10.1136/jech-2020- 213817

Totsika, V., & Sylva, K. (2004). The Home Observation for Measurement of the Environment Revisited. *Child and Adolescent Mental Health*, *9*(1), 25–35. https://doi.org/10.1046/j.1475- 357X.2003.00073.x
